# Human RNase 4 improves mRNA sequence characterization by LC–MS/MS

**DOI:** 10.1093/nar/gkac632

**Published:** 2022-07-25

**Authors:** Eric J Wolf, Sebastian Grünberg, Nan Dai, Tien-Hao Chen, Bijoyita Roy, Erbay Yigit, Ivan R Corrêa

**Affiliations:** New England Biolabs, Inc, 43/44 Dunham Ridge, Beverly, MA 01915, USA; New England Biolabs, Inc, 43/44 Dunham Ridge, Beverly, MA 01915, USA; New England Biolabs, Inc, 43/44 Dunham Ridge, Beverly, MA 01915, USA; New England Biolabs, Inc, 43/44 Dunham Ridge, Beverly, MA 01915, USA; New England Biolabs, Inc, 43/44 Dunham Ridge, Beverly, MA 01915, USA; New England Biolabs, Inc, 43/44 Dunham Ridge, Beverly, MA 01915, USA; New England Biolabs, Inc, 43/44 Dunham Ridge, Beverly, MA 01915, USA

## Abstract

With the rapid growth of synthetic messenger RNA (mRNA)-based therapeutics and vaccines, the development of analytical tools for characterization of long, complex RNAs has become essential. Tandem liquid chromatography–mass spectrometry (LC–MS/MS) permits direct assessment of the mRNA primary sequence and modifications thereof without conversion to cDNA or amplification. It relies upon digestion of mRNA with site-specific endoribonucleases to generate pools of short oligonucleotides that are then amenable to MS-based sequence analysis. Here, we showed that the uridine-specific human endoribonuclease hRNase 4 improves mRNA sequence coverage, in comparison with the benchmark enzyme RNase T1, by producing a larger population of uniquely mappable cleavage products. We deployed hRNase 4 to characterize mRNAs fully substituted with 1-methylpseudouridine (m^1^Ψ) or 5-methoxyuridine (mo^5^U), as well as mRNAs selectively depleted of uridine–two key strategies to reduce synthetic mRNA immunogenicity. Lastly, we demonstrated that hRNase 4 enables direct assessment of the 5′ cap incorporation into *in vitro* transcribed mRNA. Collectively, this study highlights the power of hRNase 4 to interrogate mRNA sequence, identity, and modifications by LC–MS/MS.

## INTRODUCTION

Significant advances in mRNA synthesis and delivery technologies have enabled the development of a breadth of mRNA-based vaccines and therapeutics that show promise to prevent or ameliorate diverse human disease conditions (1). RNA modifications are key to the efficacy of expression of *in vitro* transcribed (IVT) therapeutic mRNAs. The incorporation of specific modified uridine nucleotides, including pseudouridine (Ψ), *N*1-methylpseudouridine (m^1^Ψ) and 5-methoxyuridine (mo^5^U) have been shown to reduce immunogenicity and enhance translation and stability of exogenously delivered mRNAs (2–7). Furthermore, enzymatic capping or template-directed introduction of variants of a 7-methylguanosine 5′-triphosphate (m^7^Gppp) 5′-cap is critical for mRNA stability and translation (8). Of note, the U.S. Food and Drug Administration approved mRNA-based COVID-19 vaccine BNT162b2, whose primary sequence is known, is fully substituted with m^1^Ψ and incorporates a 3′-*O*-Me-m^7^G(5′)ppp(5′)Am 5′-cap variant (9).

Analytical tools that directly assess the fidelity and integrity of the primary sequence of an IVT mRNA of interest are critical in all phases of research, development, and production of therapeutic mRNAs. Tandem liquid chromatography–mass spectrometry (LC–MS/MS) is commonly used for verifying the identity, sequence, and purity of synthetic RNA oligonucleotides, as well as for determining the presence and position of RNA modifications (10). However, the characterization of full-length mRNAs by LC–MS/MS is technically challenging and has been hindered by a lack of robust analytical and computational tools. Instead, borrowing concepts from shotgun proteomics, mRNAs are digested with one or more site-specific endoribonucleases and the resulting cleavage products are separated by LC and analyzed by multistage MS (11–13).

Among the endoribonucleases that have been successfully utilized for characterization of long RNAs (>200 nt) include RNase T1 (G-specific), MC1 (U-specific), Cusativin (C-specific), Colicin E5 (‘GU’-specific) and *Escherichia coli* MazF (‘ACA’-specific) (13–17). Coupling endoribonuclease digestion workflows to LC–MS/MS presents several fundamental obstacles. First, the RNA secondary structure can interfere with the activity of a particular endoribonuclease. Second, endoribonucleases often produce a mixture that may contain 2′,3′-cyclic-phosphorylated, 3′-phosphorylated, and 2′,3′-hydroxylated digestion products, which convolutes and reduces the sensitivity of the analysis by mass spectrometry. Third, there are a limited number of commercially available endoribonucleases with discrete recognition and cleavage specificities that have been fully characterized, are robust and sufficiently pure to generate reliable and reproducible oligonucleotide products for downstream analysis. Fourth, the activity of many endoribonucleases is impaired by the presence of RNA modifications.

Moreover, two competing challenges exist regarding the cleavage specificities of endoribonucleases for the analysis of long RNAs. Endoribonucleases that cleave RNA at a single nucleotide (e.g. RNase T1) produce predominantly short cleavage products, which are often not uniquely mappable, thereby complicating annotation of oligonucleotide identity and reducing overall sequence coverage (13,17). Whereas endoribonucleases that cleave RNA at dinucleotide or trinucleotide sites (e.g. Colicin E5 and *E. coli* MazF) produce many lengthier cleavage products (>40 nt), which exhibit complex mass fragmentation profiles that are challenging to interpret (13). Thus, the use of combinations of endoribonucleases with distinct specificities, in parallel digestions, is a common practice to achieve adequate coverage of long RNA sequences by LC–MS/MS (13,17).

In this study, we describe the application of human RNase 4 (hRNase 4) to LC–MS/MS analysis of IVT mRNAs. hRNase 4 is one of the eight members of the human RNase A superfamily of endoribonucleases (18). Although the precise biological functions of hRNase 4 are not well understood, the hRNase 4 gene is present within a transcriptional regulatory unit together with angiogenin (hRNase 5) (19)—an angiogenic and neuroprotective factor with associations to the pathology of amyotrophic lateral sclerosis (20). Interestingly, like angiogenin, initial studies suggested that hRNase 4 can induce angiogenesis and stimulate neuronal differentiation (21), and may play a role in anti-viral response (22). Notably, the endoribonuclease activity of hRNase 4 is better understood. hRNase 4 is a uridine-specific endoribonuclease with a preference to cleave RNA after uridine residues prior to purines (UR, wherein R = A or G) (23,24). Here, we demonstrate that hRNase 4, whose cleavage specificity lies between that of mono- and dinucleotide-specific endoribonucleases, provides higher mapping coverage in comparison with other RNases used to date in LC–MS/MS-based mRNA sequencing workflows, including coverage of long mRNAs (>4000 nt). Furthermore, we show that hRNase 4 tolerates various uridine nucleobase modifications and thus can be utilized to characterize mRNAs fully substituted with 1-methylpseudouridine (m^1^Ψ) and 5-methoxyuridine (mo^5^U). Lastly, we leverage hRNase 4 to identify the presence of a 7-methylguanosine 5′-triphosphate 2′-*O*-methyladenosine (m^7^GpppAm) cap structure on an IVT mRNA. hRNase 4 is a new addition to the enzymatic toolkit for RNA analysis by mass spectrometry and provides opportunities to further expand coverage of the RNA sequence and modification status.

## MATERIALS AND METHODS

### Materials

All reagents were from New England Biolabs (NEB), Ipswich, MA, USA, unless otherwise specified. Oligonucleotides were obtained from Integrated DNA Technologies (IDT), Coralville, IA, USA; from TriLink BioTechnologies, San Diego, CA, USA; or from Bio-Synthesis Inc., Lewisville, TX, USA. The RNA oligonucleotide pools used in this study are described in Supplementary Table S1 (multiplexed pool comprising all possible dinucleotide combinations at least once) and Supplementary Table S3 (multiplexed pool comprising uridine modifications).

### Expression and purification of hRNase 4

Recombinant wild type hRNase 4 was periplasmically expressed as an MBP fusion protein containing an N-terminal signal peptide (61.7 kDa) (Supplementary Figure S1A) according to a protocol adapted from (16). Expression of hRNase 4 was induced with 10 μM IPTG from a periplasmic hRNase 4-containing plasmid in T7 Express lysY Competent *E. coli* [MiniF lysY (CamR)/fhuA2 lacZ::T7 gene1 [lon] ompT gal sulA11 R(mcr-73::miniTn10–TetS)2 [dcm] R(zgb-210::Tn10–TetS) endA1 Δ(mcrC-mrr)114::IS10] (NEB #C3010I) for 16 h at 16°C. The cells were lysed by sonication in lysis buffer (20 mM Tris–HCl pH 7.5, 200 mM NaCl, 1 mM DTT) and protease inhibitors (1 mM PMSF, 0.5 nM leupeptin, 2.75 mM benzamidine, 2 nM pepstatin), followed by the removal of cell debris by centrifugation at 21 000 × g for 1 h. The enzyme was purified from the crude extract using 10 ml BioRad Econo-Pac disposable chromatography columns (Bio-Rad Laboratories, Hercules, CA, USA) packed with 1.5 ml Amylose Resin (NEB #E8021). The flow rate during loading, washing, and elution was regulated to ∼0.8 ml/min using a Discofix^®^ 1-way stopcock. After elution in elution buffer (EB1; 20 mM Tris–HCl pH 7.5, 250 mM NaCl, 5 mM mercaptoethanol, 20 mM maltose), the protein was loaded onto a His GraviTrap Talon column (Cytiva, Marlborough, MA, USA), equilibrated with binding buffer (20 mM Na_2_HPO_4_ pH 7.5, 0.5 M NaCl, 5 mM mercaptoethanol, 5 mM imidazole). hRNase 4 was eluted in two 3 ml fractions with elution buffer (20 mM Na_2_HPO_4_ pH 7.5, 0.5 M NaCl, 5 mM mercaptoethanol, 0.5 M imidazole). The enzyme-containing fraction was dialyzed with 200 mM NH_4_OAC, pH 5.5, 1 mM DTT, and after dialysis supplemented with an equal volume of 100% glycerol. The hRNase 4 concentration (77 μg/ml, 1.31 μM) in the storage buffer (100 mM NH_4_OAC, pH 5.5, 0.5 mM DTT, and 50% glycerol) was determined by gel quantification using a BSA standard curve.

### Multiplexed assays to assess endoribonuclease cleavage specificity

The multiplexed assay to assess the dinucleotide cleavage specificity of hRNase 4 was performed as described in (16) with minor modifications. A series of 10-, 20- and 40-fold dilutions of purified hRNase 4 was prepared in NEBuffer 1 (10 mM Bis–Tris–propane–HCl pH 7, 10 mM MgCl_2_, 1 mM DTT). 2 μl of each hRNase 4 dilution were incubated with an oligonucleotide pool containing all possible dinucleotide combinations (25 pmol of each oligonucleotide) (Supplementary Table S1) in NEBuffer 1 at 37°C for 1 h in a 20 μl reaction volume with shaking at 300 rpm. Comparative experiments were performed in absence of the endoribonuclease or utilizing 2 μl of a 50-fold dilution of RNase T1 (1000 U/μl, Thermo Fischer Scientific, Waltham, MA, USA) in NEBuffer 1 at 37°C for 30 min. Each reaction mixture was subsequently filtered utilizing a Ultrafree MC-GV 0.22 um spin column (Millipore Sigma, Burlington, MA, USA) at 13 400 rpm for 5 min. The resultant digestion products were characterized by LC–MS/MS.

For analysis of the cleavage sensitivity of hRNase 4 to different uridine modifications, a pool of oligonucleotides (25 pmol of each oligonucleotide) was prepared wherein each oligonucleotide contains one putative hRNase 4 cleavage site consisting of a uridine or modified uridine (Um, Ψ, m^5^U, m^5^Um, s^4^U, D or m^1^Ψ) followed by an adenosine residue (Supplementary Table S3). The oligonucleotide pool was incubated with 2 μl of either a 1:5 dilution of hRNase 4 or a 1:50 dilution of MC1 in NEBuffer 1 at 37°C for 1 h in a 20 μl reaction volume with shaking at 300 rpm. Each reaction mixture was subsequently filtered utilizing a Ultrafree MC-GV 0.22 um spin column (Millipore Sigma) at 13 400 rpm for 5 min. The resultant digestion products were characterized by LC–MS/MS.

### Computational prediction of mRNA sequence coverages

Computational prediction of mRNA sequence coverage was performed by prediction of the theoretical digestion products for each endoribonuclease utilizing an in-house script (GitHub: https://github.com/ewolf-42/mRNA-Analysis-with-hRNase4). For this analysis, one thousand mRNA sequences were randomly selected from a database of annotated human mRNA sequences (NCBI Reference Sequence Database, RefSeq (25)). Each of those sequences was completely cleaved *in-silico* utilizing the endoribonuclease cleavage specificities listed in Supplementary Table S2. Only contiguous cleavage products between 4 and 40 nucleotides (nt) in length were considered for downstream analysis. Each cleavage product with a unique monoisotopic mass was annotated as ‘uniquely mappable’ and cleavage products with a non-unique monoisotopic mass were annotated as ‘isomeric’. Cleavage products with a non-unique sequence were excluded from downstream analysis. The predicted coverage for each mRNA sequence was calculated by summing the lengths of the cleavage product produced by each endoribonuclease and then dividing it by the mRNA length, using either all cleavage products or only the uniquely mappable cleavage products.

### mRNA synthesis

mRNAs were synthesized by *in vitro* transcription (IVT) reaction utilizing the HiScribe™ T7 High Yield RNA Synthesis Kit (NEB # E2050S) with the respective linearized plasmid DNA templates. Template sequences can be found in Supplementary Table S4. A 20 μl solution of 1 μg of linearized DNA template, 10 mM of each ribonucleic acid triphosphate (NTP), 2 μl of T7 RNA Polymerase Mix in 1× T7 Reaction Buffer was incubated at 37°C for 2 h. Following incubation, 10 μl of 10× DNase I Reaction Buffer (1× Reaction Buffer: 10 mM Tris–HCl pH 7.6, 2.5 mM MgCl_2_, 0.5 mM CaCl_2_) and 2 μl of DNase I (or 1 μl DNase Turbo, Thermo Fisher Scientific) were added to the mixture. The solution was incubated at 37°C for 15 min to digest the template DNA strand utilized for mRNA synthesis. Alternatively, Turbo DNase (Thermo Fisher Scientific) was added directly to the reaction and incubated at 37°C for 30 min for DNA digestion. After DNase digestion, the mRNA was purified utilizing Monarch^®^ RNA Cleanup Kit (500 μg) (New England Biolabs, Cat # T2050S). RNA quantities were determined on a Nanodrop (Thermo Fischer Scientific) UV spectrophotometer. For transcription of m^1^Ψ and mo^5^U-modified mRNA, uridine-triphosphate was substituted with m^1^Ψ-triphosphate or mo^5^U-triphosphate (TriLink BioTechnologies) in each IVT reaction, other procedures were identical to described above. Co-transcriptional m^7^GpppAm capping was performed utilizing the CleanCap^®^ AG system (TriLink BioTechnologies) (26).

### mRNA endoribonuclease digestion

Each mRNA (3–10 μg; see Supplementary Table S4) was mixed in a denaturing solution of 3 M urea in NEBuffer 1. To denature mRNA structure, the sample was incubated at 90°C for 10 min and quickly cooled to room temperature. The cooled mRNA solution was diluted threefold in NEBuffer 1. For hRNase 4 digestion, between 1 and 3 μl of hRNase 4 and 160 units of T4 PNK (New England Biolabs) were added to the diluted mRNA mixture and incubated at 37°C for 2 h with shaking at 300 rpm. For RNase T1 digestion, 1 μl of RNase T1 (Thermo Fischer Scientific) was added to the diluted mRNA mixture and incubated at 37°C for 1 h with shaking at 300 rpm. The resultant digests were filtered utilizing a Ultrafree MC-GV 0.22 μm spin column (Millipore Sigma) at 13 400 rpm for 5 min.

### RNA oligonucleotide LC–MS/MS

LC–MS/MS was performed according to a protocol modified from (16). Ultra-high-performance liquid chromatography (UHPLC) separation of RNA oligonucleotides was performed on a Thermo Scientific Vanquish Horizon UHPLC system equipped with a DNAPac™ RP Column (2.1 × 50 mm, 4 mm) at 70°C utilizing a 25-min 5–35% gradient of solvent A (1% hexafluoroisopropanol (HFIP), 0.1% *N*,*N*-diisopropylethylamine (DIEA), 1 μM EDTA) and increasing solvent B (80% Methanol, 0.075% HFIP, 0.0375% DIEA, 1 μM EDTA) at a 300 μl/min flow rate. High-resolution mass spectrometry was performed on a Thermo Scientific Q Exactive Plus orbitrap mass spectrometer operating under negative electrospray ionization mode (–ESI). Tandem mass spectrometry (MS/MS) analysis was performed in data-dependent acquisition mode (ddMS^2^). Oligonucleotide MS^1^mass data was collected at a resolution of 70 000 (FWHM) at *m/z* 200. The top-5 masses in each oligonucleotide MS^1^ mass spectrum (with dynamic exclusion) were subjected to HCD-fragmentation at a resolution of 35 000 (FWHM) and a normalized collision energy of 20%.

### Oligonucleotide MS^1^ mass data analysis

Oligonucleotide MS^1^ mass data was charge deconvoluted utilizing ProMass HR (Novatia LLC) and the Avalon peak picking algorithm (Thermo Fischer Scientific). ProMass HR employs a ‘ZNova’ deconvolution algorithm for charge-state deconvolution, which is based on the ‘component deconvolution’ approach as described in (27). For analysis of uniquely mapped cleavage products, the deconvoluted masses were compared with the predicted masses obtained from theoretical digestion of each mRNA sequence with the endoribonuclease using a mass difference cutoff of 10 ppm and a maximum of one missed cleavage. In the case of multiple masses matching to a single sequence, the intensity was summed for downstream analysis.

For mRNA identity analysis, deconvoluted masses detected in each experiment were compared to those of cleavage products obtained from a complete theoretical digestion of the RefSeq database of human transcripts supplemented with a target mRNA(s)-of-interest. A 5-ppm mass difference cutoff was set for each comparison. Both unique mappable and isomeric products were included in all calculations. The ‘identity score’ for each transcript was defined as the product of the fraction of total spectral intensity explained by the matched cleavage products and the fraction of theoretical cleavage products detected in the mass spectrum. mRNA identity analysis was performed utilizing an in-house script that is available at (Github: https://github.com/ewolf-42/mRNA-Analysis-with-hRNase4). Signal-to-noise ratios (S/N) were computed for each digest from the identity score of the known mRNA input divided by the standard deviation of the identity scores of all other human transcripts.

For the analysis of mRNA 5′ m^7^GpppAm capping, the theoretical masses of 5′ terminal EPO mRNA hRNase 4 cleavage products with up to two missed cleavages from the 5′ end were compared to the deconvoluted oligonucleotide MS^1^ mass data from each experiment. Variable addition of monophosphate, diphosphate, triphosphate, methyl guanosine triphosphate and dimethyl guanosine triphosphate masses were included to identify putative capped 5′ terminal cleavage products.

### MS/MS data analysis

Tandem MS/MS data analysis was performed utilizing the Nucleic Acids Search Engine (NASE) (28) in Open-MS (version: 2.6.0) (29). Precursor and fragment ion mass cutoffs of 3 ppm were utilized. Na^+^/K^+^ adduct precursor masses, and 0 to +4 precursor isotopes between the charge states –1 to –20 were considered for analysis. Fragment ions as defined by (30) (a-B, a, b, c, d, w, x, y and z) were considered for analysis of tandem mass spectra. MS/MS data were searched against theoretical digests of the appropriate mRNA sequence with either RNase T1 or hRNase 4 with up to two missed cleavages. Only 3′-phosphorylated or 2′,3′-hydroxylated cleavage products were considered in RNase T1 or hRNase 4/T4 PNK digests, respectively. For comparative analysis of digestion with hRNase 4 in the presence or absence of T4 PNK, 3′-phosphorylated, 2′,3′-cyclicphosphorylated and 2′,3′-hydroxylated cleavage products were all considered. A target/decoy false discovery rate (FDR) utilizing shuffled oligonucleotides of 5% was applied. Exact duplicate oligonucleotides were removed for downstream sequence coverage calculations.

## RESULTS

### hRNase 4 cleaves RNA primarily after U prior to purines

To investigate the utility of hRNase 4 for RNA LC–MS/MS workflows, we expressed and purified a recombinant maltose binding protein (MBP)-tagged hRNase 4 (MBP-hRNase 4) fusion protein, according to a previously described *E. coli* expression platform (16) (Supplementary Figure S1A). To confirm the reported uridine specificity (UR) of hRNase 4 (23), we assessed its cleavage pattern in a multiplexed pool of oligonucleotides containing all possible dinucleotide combinations (present at least once) flanked by a poly-adenosine backbone (Supplementary Table S1) (16). We benchmarked our approach by incubating the multiplexed oligonucleotide pool with the well-characterized guanosine-specific endoribonuclease RNase T1 (31). Consistent with the expected guanosine specificity, we observed robust cleavage of all oligonucleotides in our pool containing at least one guanosine residue (Supplementary Figure S1B).

**Figure 1.**
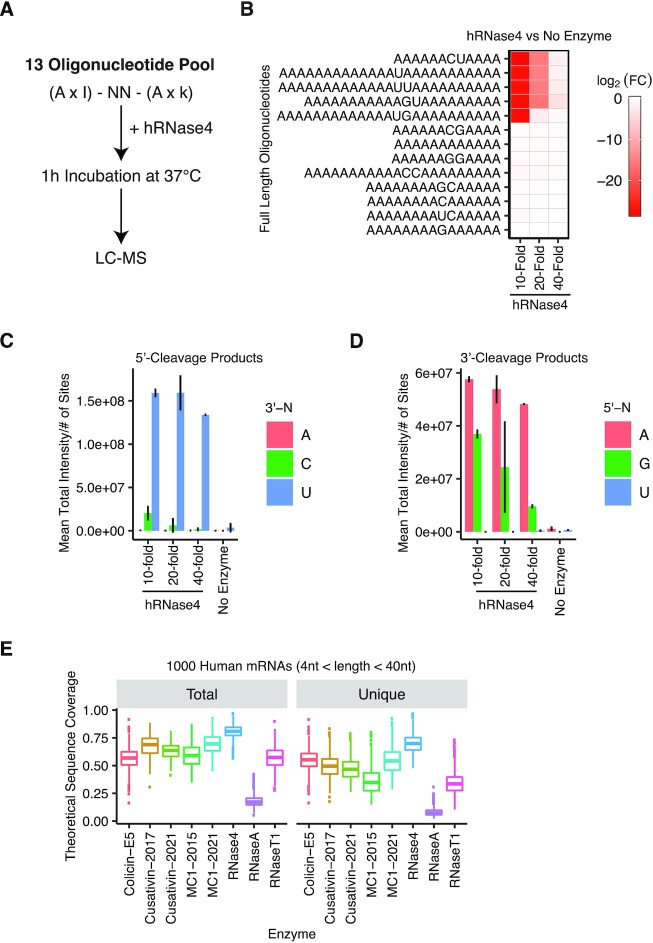
hRNase 4 cleaves RNA primarily downstream of U and upstream of purines. (**A**) A schematic of the multiplexed assay to assess hRNase 4 dinucleotide cleavage specificity by LC–MS/MS. (**B**) The mean log_2_ fold intensity change for each input oligonucleotide in a multiplexed oligonucleotide pool (Supplementary Table S1) after incubation with a dilution series of hRNase 4 relative to experiments performed in the absence of the enzyme. Results are from two independent experiments. (**C**) The mean total intensity of 5′ cleavage products normalized to the number of possible sites produced upon incubation of the multiplexed oligonucleotide pool with a dilution series of hRNase 4 binned by the 3′ terminal nucleotide (3′-N). Error bars represent standard deviation from two independent experiments. (**D**) The mean total intensity of 3′ cleavage products normalized to the number of possible sites produced upon incubation of the multiplexed oligonucleotide pool with a dilution series of hRNase 4 binned by the 5′ terminal nucleotide (5′-N). Error bars represent standard deviation from two independent experiments. (**E**) The distribution of sequence coverages of total and uniquely mappable cleavage products between 4 and 40 nt in length obtained from complete theoretical digestion of 1000 randomly selected human mRNAs (RefSeq) of less than 5 kB in size. The cleavage specificities of the endoribonucleases used in this study are listed in Supplementary Table S2.

To test the specificity of recombinant hRNase 4, we incubated the multiplexed oligonucleotide pool with a dilution series of the enzyme (Figure 1A). We observed cleavage of most oligonucleotides containing at least one uridine residue, with clear exception of the ‘UC’-containing sequence (Figure 1B; see Supplementary S1C for a representative chromatogram). To better define the specificity of hRNase 4, we examined each of the individual 5′ and 3′ cleavage products (Figure 1C and D). The 5′ cleavage products predominantly exhibited a 3′ terminal uridine (Figure 1C) and were a mixture of 2′,3′-cyclic-phosphorylated and 3′-phosphorylated species (Supplementary Figure S1D), with the former being more predominant at low hRNase 4 concentrations. Notably, some very low levels of 5′ cleavage products with a 3′-terminal cytidine were detected (10- to 50-fold lower relative abundance), particularly at higher hRNase 4 concentrations (Figure 1C). Lastly, we observed 3′ cleavage products predominantly with a 5′ terminal adenosine and to a lesser extent a 5′ terminal guanosine, confirming previous reports that hRNase 4 preferably cleaves uridine prior to purine residues (Figure 1D), and further indicating that the addition of an MBP tag has no effect on hRNase 4 specificity.

We then compared *in silico* how the UR cleavage specificity impacts the sequence coverage of long RNAs relative to that of other endoribonucleases previously used in LC–MS/MS workflows (see Supplementary Table S2 for a list of the endoribonucleases used in this study). To this end, we performed a theoretical digestion of 1000 randomly selected human mRNA transcript sequences (<5 kB) utilizing the reported specificity for each endoribonuclease. Subsequently, we calculated the predicted transcript sequence coverage considering all possible cleavage products generated ranging from 4 to 40 nt in length (this oligonucleotide range was chosen because it is amenable for both oligonucleotide MS^1^ mass and MS/MS analyses) and excluding any cleavage products with non-unique sequences. hRNase 4 produced the largest number of RNA cleavage products with a unique monoisotopic mass (hereafter referred to as ‘uniquely mappable’) among all entered endoribonucleases, thus resulting in the highest theoretical sequence coverage (Figure 1E). While in practice the actual sequence coverage may vary as the cleavage efficiency depends on the reaction conditions (such as buffer composition, pH, salt concentration, temperature, incubation time, and enzyme concentration), the presence of secondary structures and/or RNA modifications, and the quality of the endoribonuclease preparation (such as the presence of contaminating nucleases or absence of cofactors), hRNase 4 appeared especially suitable for analysis of mRNA and other long RNA substrates.

### hRNase 4 improves mRNA sequence validation by LC–MS/MS

**Figure 2.**
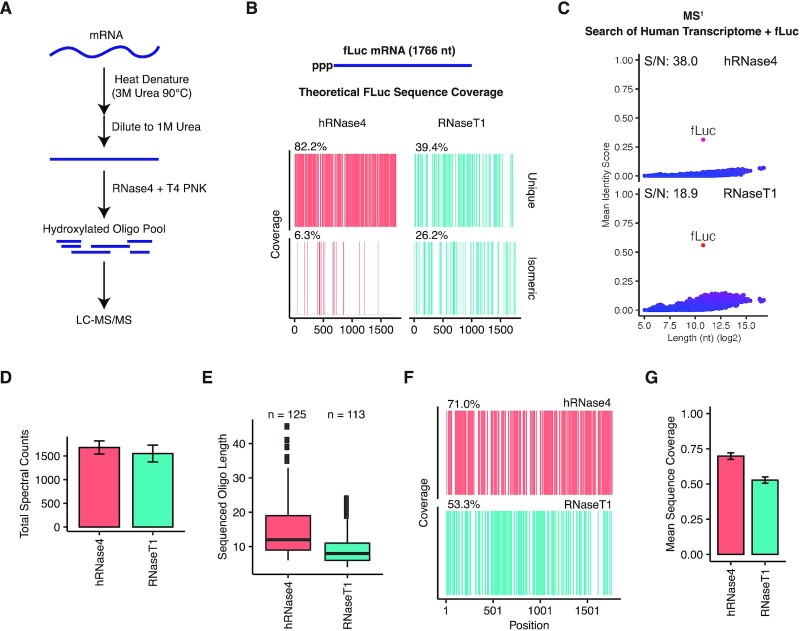
hRNase 4 improves mRNA sequence validation by LC–MS/MS. (**A**) A schematic of the digestion method combining hRNase 4 and T4 PNK to characterize mRNA by LC–MS/MS. (**B**) A sequence coverage map of the uniquely mappable and isomeric cleavage products greater than 4 nt from complete theoretical digestion of fLuc mRNA with hRNase 4 and RNase T1. (**C**) A search of the deconvoluted oligonucleotide MS^1^ masses detected in hRNase 4 and RNase T1 digests of fLuc mRNA against predicted cleavage product masses from annotated human transcripts (RefSeq database) supplemented with the fLuc mRNA (see Materials and Methods). The mean identity score from three fLuc mRNA digestions is reported for each transcript. The mean signal-to-noise ratio (S/N) of the fLuc mRNA sequence score relative to all other transcripts is reported at the top of each graph. (**D**) The mean total number of tandem MS/MS spectra mappable to hRNase 4 or RNase T1 fLuc mRNA cleavage products as determined by NASE (5% FDR). Error bars represent standard deviation from three independent experiments. (**E**) The length distribution of the sequenced cleavage products detected by NASE from digestion of fLuc mRNA with either hRNase 4 or RNase T1. The total number of distinct oligonucleotides detected from three independent experiments are reported above each boxplot. (**F**) A sequence coverage map of positions in the fLuc mRNA detected in at least two independent digestions with either hRNase 4 or RNase T1. The percent sequence coverage is reported above each map. (**G**) The mean fractional coverage of fLuc mRNA sequence obtained from hRNase 4 or RNase T1 sequenced cleavage products. Error bars represent standard deviation from three independent experiments.

To assess the experimental utility of hRNase 4 for mRNA analysis, we first developed a method that streamlines the digestion process and reduces the complexity of the product oligonucleotides prior to downstream LC–MS/MS analysis (Figure 2A). This method involved heat denaturing the mRNA in the presence of urea to relax any secondary structures, followed by a 3-fold dilution to reduce the content of the denaturing agent, and subsequent treatment (without any purification steps) with hRNase 4 and T4 polynucleotide kinase (T4 PNK). T4 PNK is capable of dephosphorylating both 2′,3′-cyclic-phosphorylated and 3′-phosphorylated oligoribonucleotides, and in the absence of ATP does not produce 5′-phosphorylated products (32). We found that hRNase 4 and T4 PNK can be used concurrently in the same reaction buffer with up to 1 M urea without significant loss of activity (data not shown). Addition of T4 PNK to the digestion reaction resolved the mixture of phosphorylated products by yielding uniformly hydroxylated oligonucleotide termini (Supplementary Figure S2A).

We applied our method to a 1,766 nt IVT mRNA encoding firefly luciferase (fLuc). Complete digestion of fLuc mRNA with hRNase 4 was computationally predicted to yield a maximum of ∼82% coverage using uniquely mappable oligonucleotides compared to ∼39% with RNase T1 (Figure 2B). fLuc mRNA was experimentally digested with either hRNase 4 supplemented with T4 PNK (as shown in Figure 2A) or RNase T1, then analyzed by high-resolution orbitrap-based LC–MS/MS. We first analyzed the cleavage products matching the deconvoluted masses detected by oligonucleotide MS^1^ mass analysis, considering cleavage products with a maximum of one missed cleavage event (see Materials and Methods for details). hRNase 4 produced a substantially higher number of longer, uniquely mapping cleavage products in comparison with RNase T1 (Supplementary Figure S2B and C). Consistent with our computational prediction, this resulted in a higher fraction of the fLuc mRNA sequence that was uniquely mapped with cleavage products from hRNase 4 reaction (∼78%) relative to those from RNase T1 (∼40%) (Supplementary Figure S2D and E). It is noteworthy that such high mapping coverage is primarily attributable to oligonucleotides resulting from complete fLuc mRNA digestion with hRNase 4, i.e. with no missed cleavages (Supplementary Figure S2F). Oligonucleotide products resulting from a single missed cleavage event were observed at a reduced intensity and frequency relative to those from a complete digestion (Supplementary Figures S2G and H). Most missed cleavage events were observed at ‘UG’ sites (Supplementary Figure S2I), which is in line with the slight preference of hRNase 4 for cleavage at ‘UA’ relative to ‘UG’ sites (Figure 1D).

Given the robust sequence coverage obtained for fLuc mRNA with hRNase 4, we asked whether the profile of deconvoluted masses could be utilized as a ‘mass fingerprint’ to verify the identity of a given mRNA in the context of a larger sequence database. Briefly, we compared each mass profile against the mass profiles obtained from a complete theoretical digestion of all annotated human transcripts from the RefSeq database, supplemented with the fLuc mRNA sequence. The product of the proportion of total intensity explained by cleavage products and the proportion of all cleavage products identified from each transcript was calculated, hereafter referred to as the ‘identity score’ (see Materials and Methods for details). Using this approach, fLuc mRNA exhibited the highest identity score in a search of all deconvoluted oligonucleotide MS^1^ masses generated by either hRNase 4 or RNase T1 against the ‘spiked’ database, consistent with the high specificity of both endoribonucleases (Figure 2C). The identity score background was higher for RNase T1 resulting in a lower signal-to-noise ratio, likely due to a higher proportion of shared cleavage products among transcripts relative to those of hRNase 4.

Next, we sequenced the oligonucleotide products in each experiment by tandem MS/MS utilizing high-energy collisional dissociation (HCD) to generate high resolution fragments of intact precursor ions. To identify candidate oligonucleotide sequences mapping to each MS/MS spectra, we searched the data using the Nucleic Acid Search Engine (NASE) in OpenMS (28) with a 5% false discovery rate (FDR) cutoff and considering cleavage products with a maximum of two missed cleavage events (see Methods for details). A similar number of sequenced cleavage products were identified in hRNase 4 and RNase T1 digests (Figure 2D). Consistent with the results from oligonucleotide MS^1^ mass analysis, the sequenced cleavage products that were identified following digestion with hRNase 4 were longer (Figure 2E) and led to a higher coverage of the fLuc mRNA sequence (∼71%) than those identified with RNase T1 (∼53%) (Figure 2F and G). It is worth noting that there was a slight decrease in RNA sequence coverage with hRNase 4 through fragmentation analysis (∼71%) (Figure 2F and G) compared to oligonucleotide MS^1^ mass analysis (∼78%) (Supplementary Figures S2D and E). This could be attributed to a subset of cleavage products that either were too long for interpretation by the NASE algorithm, not selected for fragmentation, or not otherwise identified by MS/MS-based oligonucleotide sequencing.

To verify that the improvement in fLuc sequence coverage upon digestion with hRNase 4 in combination with T4 PNK was primarily due to the activity of the endoribonuclease, we performed a series of fLuc mRNA digestion experiments in the presence and absence of T4 PNK. Co-incubation of T4 PNK with hRNase 4 simplified the mixture of diversely phosphorylated cleavage products (Supplementary Figure S2J) and improved the quality of spectra sequenced by MS/MS (NASE score) (Supplementary Figure S2K). However, no difference in the overall sequence coverage was observed between digestions in the presence and absence of T4 PNK (Supplementary Figure S2L). Taken together, the activity and specificity of hRNase 4 are primarily responsible for rendering a population of longer and uniquely mappable cleavage products, and thus substantially improving mRNA sequence coverage by both oligonucleotide MS^1^ mass analysis and fragmentation-based oligonucleotide sequencing.

### hRNase 4 discriminates between mRNAs fully modified with m^1^Ψ and mo^5^U

**Figure 3.**
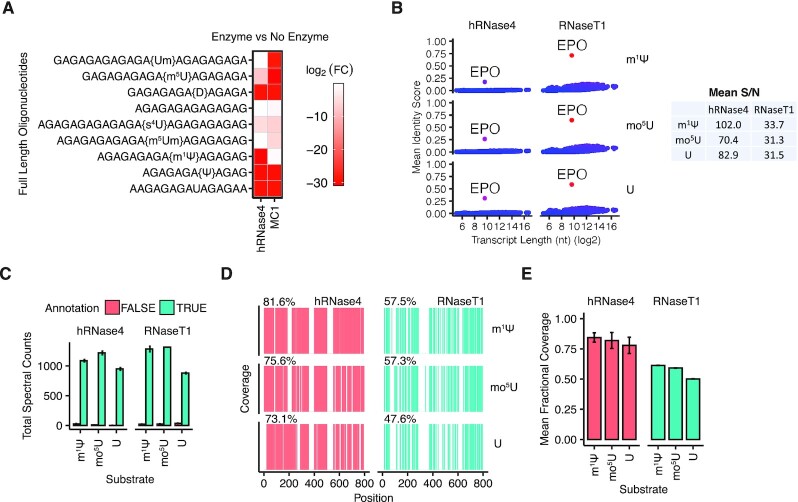
hRNase 4 discriminates between mRNAs modified with m^1^Ψ and mo^5^U. (**A**) The mean log2 fold intensity change of oligonucleotides in a multiplexed pool comprising uridine modifications (Supplementary Table S3) after incubation with either hRNase 4 or MC1 relative to the corresponding conditions in the absence of an endoribonuclease. Results are from two independent experiments. (**B**) (Left) A search of the deconvoluted oligonucleotide MS^1^ masses detected in hRNase 4 or RNase T1 digests of U-, m^1^Ψ- or mo^5^U-modified EPO mRNA against predicted cleavage product masses from annotated human transcripts (RefSeq database) supplemented with the EPO mRNA (see Methods). U-, m^1^Ψ- or mo^5^U-modified RNA sequences were utilized for each corresponding search. The mean identity score was calculated from two independent experiments as described in Figure 2C. (Right) The mean signal-to-noise ratio (S/N) of the score of each U-, m^1^Ψ- or mo^5^U-modified EPO mRNA sequence relative to all other transcripts. (**C**) The mean total number of sequenced cleavage products in hRNase 4 or RNase T1 digests of U-, m^1^Ψ- or mo^5^U-modified EPO mRNA (5% FDR). Cleavage products are grouped by true (green) and false (red) assignments to a modified EPO mRNA. Error bars represent standard deviation from two independent experiments. (**D**) A sequence coverage map of positions in the U-, m^1^Ψ- or mo^5^U-modified EPO mRNA detected in two independent digestions with either hRNase 4 or RNase T1. The percent sequence coverage is reported above each map. (**E**) The mean fractional coverage of the U-, m^1^Ψ- or mo^5^U-modified EPO mRNA sequences obtained from hRNase 4 or RNase T1 sequenced cleavage products. Error bars represent standard deviations from two independent experiments.

One of the primary advantages of LC–MS/MS for characterization of mRNA is the capability to directly detect and map RNA modifications. Prophylactic and therapeutic mRNAs are often fully substituted with modified uridines, such as m^1^Ψ or mo^5^U, to reduce their immunogenicity and improve stability and translation properties (2–7). To test the sensitivity of hRNase 4 to uridine modifications, we assessed the cleavage of a multiplexed pool of oligonucleotides (with a poly-AG backbone), each containing one putative hRNase 4 cleavage site consisting of a modified uridine followed by an adenosine residue (Figure 3A) (see Supplementary Table S3 for oligonucleotide sequences). hRNase 4 efficiently cleaved oligonucleotides containing uridine nucleobase modifications Ψ, m^1^Ψ and dihydrouridine (D), and to a lesser extent 4-thiouridine (s^4^U) and 5-methyluridine (m^5^U) (Figure 3A). hRNase 4 did not cleave oligonucleotides containing uridine ribose modifications, including 2′-*O*-methyluridine (Um) and 5,2′-*O*-dimethyluridine (m^5^Um) (Figure 3A), which is consistent with the formation of a 2′,3′-cyclic-phosphate ester intermediate during the ribonuclease-catalyzed RNA hydrolysis (Supplementary Figure S1D). Notably, MC1, which is another uridine specific ribonuclease (14,16,17), did not cleave the m^1^Ψ-modified oligonucleotide, but cleaved oligonucleotides containing Um and m^5^Um (Figure 3A). While not surprising, given that MC1 cuts prior to uridine residues (and thus is not likely affected by the presence of uridine 2′-*O*-methylation), these results highlight the need for a breadth of endoribonucleases to interrogate RNA substrates harboring different modifications.

Next, we tested whether hRNase 4 could be utilized to characterize and discriminate between IVT mRNAs fully substituted with m^1^Ψ or mo^5^U. To this end, we chose an 800 nt mRNA encoding Erythropoietin (EPO) as a model substrate, which has been previously employed for proof-of-concept mRNA-based interventions to treat anemia (33,34), as well as for mRNA LC–MS/MS sequence validation (13). EPO mRNA fully substituted with m^1^Ψ, mo^5^U or U was digested with either hRNase 4 or RNase T1, and the resulting products were analyzed by LC–MS/MS. EPO mRNA cleavage products from both hRNase 4 and RNase T1 reactions yielded an oligonucleotide MS^1^ mass fingerprint that was uniquely identifiable in the context of a human transcriptome database (Figure 3B). Next, we searched the tandem MS/MS data from each digest for putative hRNase 4 and RNase T1 cleavage products from U, m^1^Ψ or mo^5^U substituted EPO mRNA sequences. The vast majority of cleavage products detected in each experiment were correctly assigned to the appropriate modified substrates (Figure 3C). Concordant with our previous observations, hRNase 4 improved the sequence coverage of modified or unmodified EPO mRNAs (m^1^Ψ: 81.6%, mo^5^U: 75.6%, and U: 73.1%) relative to RNase T1 (m^1^Ψ: 57.5%, mo^5^U: 57.3% and U: 47.6%) (Figure 3D and E). Similarly, an even greater gain in sequence coverage was seen through oligonucleotide MS^1^ mass analysis of uniquely mappable sequences (Supplementary Figure S3A). Interestingly, an increase in the number of cleavage products with one missed cleavage was observed for m^1^Ψ-modified mRNA substrates (Supplementary Figure S3B), possibly due to m^1^Ψ-induced stabilization of mRNA secondary structures (35) and/or a slight reduction of hRNase 4 activity at m^1^Ψ sites.

### hRNase 4 discriminates between uridine-depleted mRNAs

**Figure 4.**
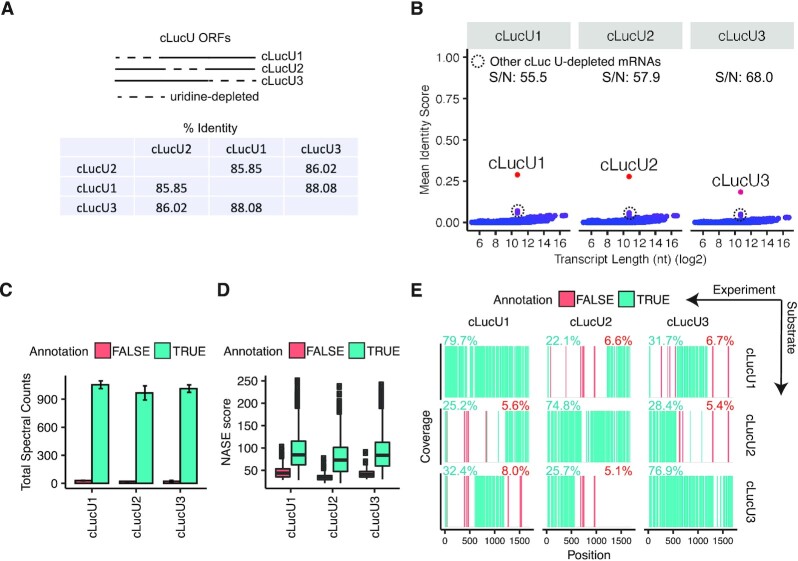
hRNase 4 discriminates between uridine-depleted mRNAs. (**A**) (Top) A schematic of the three uridine-depleted cLuc mRNAs used in this study. (Bottom) A table of the percent identity between the three uridine-depleted cLuc mRNAs (Clustal Omega). (**B**) A search of the deconvoluted oligonucleotide MS^1^ masses detected in hRNase 4 digests of the three uridine-depleted cLuc mRNAs against predicted cleavage product masses from annotated human transcripts (RefSeq database) supplemented with each uridine-depleted cLuc mRNA. The mean identity score was calculated from two independent experiments as described in Figure 2C. The mean signal-to-noise ratio (S/N) of the score of each uridine-depleted cLuc mRNA sequence relative to all other transcripts is reported at the top of each graph. (**C**) The mean total number of sequenced cleavage products in hRNase 4 digests of three uridine-depleted cLuc mRNAs (5% FDR). Cleavage products are grouped by true (green) and (red) false positive assignments to uridine-depleted cLuc mRNAs. Error bars represent standard deviation from two independent experiments. (**D**) The distribution of search engine (NASE) scores of true (green) and false (red) assigned cleavage products to each uridine-depleted cLuc mRNA detected in two independent digestions with hRNase 4. (**E**) A sequence coverage map of positions in each uridine-depleted cLuc mRNA from two independent digestions with hRNase 4. The percent sequence coverage of true (green) and false (red) assigned cleavage products are reported above each map.

In addition to uridine modification, a complementary strategy to reduce the immune response to exogenously delivered mRNAs is to selectively deplete the number of uridines present within an mRNA sequence, referred to as ‘uridine-depletion’ (36). We tested whether hRNase 4 could discriminate between three 1,703 nt mRNA sequences derived from cypridina luciferase (cLuc) that were selectively depleted of uridine in particular sub-regions (Figure 4A and Supplementary Table S4). Despite the similarity among these sequences, the oligonucleotide MS^1^ masses generated from each hRNase 4 digested uridine-depleted cLuc mRNA could be correctly annotated, with the matching uridine-depleted cLuc mRNA exhibiting the highest identity score (Figure 4B).

Next, we examined the tandem MS/MS data of each digested uridine-depleted cLuc mRNA. Most of the sequenced oligonucleotides were correctly attributed to the appropriate uridine-depleted cLuc mRNA sequence (Figure 4C). In addition, incorrectly assigned oligonucleotides were of substantially lower NASE spectral score (Figure 4D) and spectral counts (Supplementary Figure S4). Robust sequence coverage of oligonucleotides associated with the correct uridine-depleted substrate were obtained in each hRNase 4 digest (cLucU1: 79.7%; cLucU2: 74.8%; cLucU3: 76.9%) (Figure 4E). Notably, other uridine-depleted substrates exhibited some sequence coverage most of which was primarily due to overlapping cleavage products between the uridine-depleted cLuc mRNAs (Figure 4E). Collectively, these results demonstrate that hRNase 4 can distinguish between mRNA substrates with high confidence, even among highly similar sequences.

### Characterization of the BNT162b2 vaccine sequence using hRNase 4

**Figure 5.**
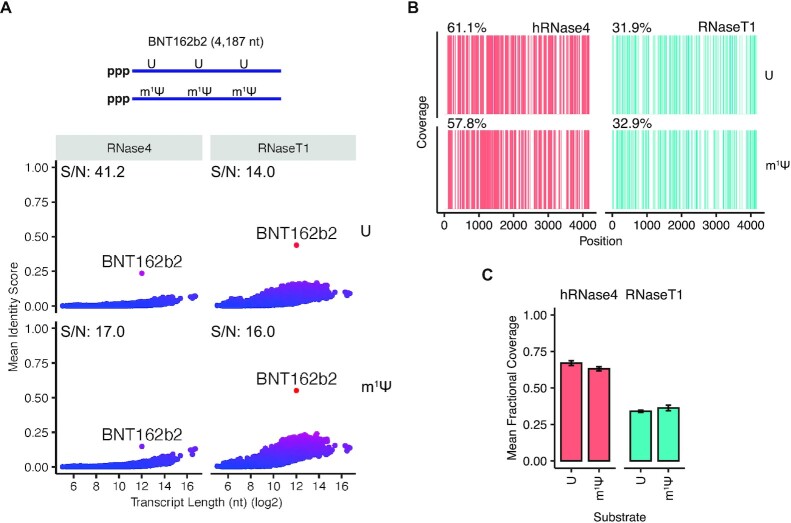
Characterization of a 4187 nt mRNA encoding the BNT162b2 sequence with hRNase 4. (**A**) (*Top*) A schematic the BNT162b2 mRNA sequence unmodified or modified with m^1^Ψ. (*Bottom*) A search of the deconvoluted oligonucleotide MS^1^ masses detected in hRNase 4 or RNase T1 digests of U- or m^1^Ψ-modified BNT162b2 mRNA against predicted cleavage product masses from annotated human transcripts (RefSeq database) supplemented with the BNT162b2 mRNA sequence. The mean identity score was calculated from two independent experiments as described in Figure 2C. The mean signal-to-noise ratio (S/N) of the BNT162b2 mRNA sequence score relative to all other transcripts is reported at the top of each graph. (**B**) A sequence coverage map of positions in the U- or m^1^Ψ-modified BNT162b2 mRNA sequence detected by NASE in two independent digestions with hRNase 4 or RNase T1. The percent sequence coverage is reported above each map. (**C**) The mean fractional coverage of the U- or m^1^Ψ-modified BNT162b2 mRNA sequence obtained from hRNase 4 or RNase T1 sequenced cleavage products. Error bars represent standard deviations from two independent experiments.

Achieving robust sequence coverage of long mRNA sequences (>4000 nt) by LC–MS/MS analysis is challenging because of the increased sequence complexity and redundancy of resulting cleavage products (13). We tested whether we could assess the identity, sequence, and modifications of a 4187 nt mRNA encoding the primary sequence (without the poly-A tail) of the BNT162b2 COVID-19 vaccine (9). Unmodified or m^1^Ψ-modified BNT162b2 was digested with either hRNase 4 or RNase T1 and analyzed by LC–MS/MS. Notably, an increased ratio of hRNase 4 (up to threefold) was necessary to achieve robust digestion of modified BNT162b2, possibly owing to its slightly reduced activity towards m^1^Ψ and an increased stabilization of mRNA structure, as noted earlier. Both hRNase 4 and RNase T1 yielded a population of cleavage products that permitted unique identification of the BNT162b2 primary sequence by oligonucleotide MS^1^ mass analysis (Figure 5A). Consistent with our fingerprinting analysis of other mRNAs, hRNase 4 provided a higher signal-to-noise ratio (S/N) of the mean identity score associated with BNT162b2 (see **Methods** for details) than RNase T1 did, particularly for the unmodified sequence (Figure 5A). More importantly, the sequence coverage of BNT162b2 produced by hRNase 4 (m^1^Ψ: 61.6% and U: 57.9%) was nearly twice that of RNase T1 (m^1^Ψ: 31.9% and U: 32.9%) (Figure 5B and C). Taken together, our data indicate that hRNase 4 can improve the characterization of long mRNA substrates (>4000 nt), which are generally challenging to assess with conventional enzymatic tools.

### Detection of an m^7^GpppAm cap structure in IVT RNAs using hRNase 4

**Figure 6.**
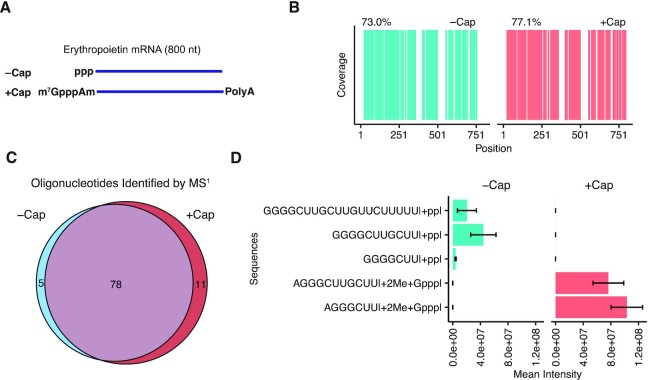
hRNase 4 enables characterization of an m^7^GpppAm capped mRNA. (**A**) A schematic of the capped and poly-adenylated EPO mRNA. (**B**) A sequence coverage map of positions in either uncapped or capped EPO mRNA detected in at least two independent digestions with hRNase 4. The percentage sequence coverage is reported above each map. (**C**) A comparison of hRNase 4 cleavage products identified by oligonucleotide MS^1^ mass analysis of uncapped and capped EPO mRNA. hRNase 4 cleavage products identified in two independent experiments are reported. (**D**) The mean intensity of 5′ terminal hRNase 4 cleavage products with a maximum of three missed cleavages from the 5′ end and variable addition of possible terminal cap structures (see Materials and Methods for details). Error bars represent standard deviation from two independent experiments.

The 5′ terminal cap structure is critical to mRNA stability and translation. We asked whether hRNase 4 could provide evidence of 5′ terminal capping in mRNA. A m^7^GpppAm cap was introduced into a preparation of EPO mRNA using a co-transcriptional capping system (26) (Figure 6A). The m^7^GpppAm-capped EPO mRNA was digested with hRNase 4 and then analyzed by LC–MS/MS. The mapping coverage of m^7^GpppAm-capped EPO mRNA obtained from analysis of all sequenced cleavage products identified by NASE (77.1%) was similar to that of uncapped EPO mRNA (73.0%) (Figure 6B). Furthermore, most oligonucleotides identified by oligonucleotide MS^1^ mass analysis were shared between hRNase 4 digests of capped and uncapped EPO mRNAs (Figure 6C). To specifically examine mRNA cap incorporation, we searched for oligonucleotide MS^1^ masses corresponding to all possible 5′ terminal cleavage products and variable addition of possible terminal cap structures (see Materials and Methods for details). We detected a series of 5′ terminal cleavage products with an increase in mass corresponding to a guanosine triphosphate and two methyl groups in the capped EPO mRNA sample, consistent with presence of a m^7^GpppAm structure (Figure 6D). Interestingly, we observed 5′ terminal diphosphorylated products in the uncapped EPO mRNA samples (Figure 6D), possibly due to the monophosphatase activity of T4 PNK. Studies to deploy hRNase 4 to accurately quantitate capping levels in IVT mRNAs are currently underway.

## DISCUSSION

hRNase 4 is a new enzymatic tool for analytical characterization of mRNAs by LC–MS/MS. Our systematic study shows that this endoribonuclease delivers a unique profile of predominantly mappable cleavage products, resulting in robust sequence coverage of mRNAs as long as 4000 nt. hRNase 4 can be utilized to identify key features relevant to medicinal mRNA applications, including the presence of modified uridines (such as m^1^Ψ and mo^5^U), targeted uridine-depletion, and 5′ capping. Notably, hRNase 4 offers an improvement on existing enzymes utilized for the sequence validation of IVT mRNAs. A recent study (13), reported sequence coverages of 73–87% for three synthetic mRNA substrates through a combination of three endoribonucleases (MazF, Colicin-E5 and RNase T1). In the present study, we showed that similar sequence coverages were achievable with hRNase 4 alone (71–81%) for mRNAs of similar sizes. Unlike these endoribonucleases, hRNase 4 produces a population of cleavage products that are of optimal length distribution for both MS/MS-based oligonucleotide sequencing and oligonucleotide MS^1^ mass analysis, thereby aiding the identification of individual oligonucleotide fragments.

Given the robust sequence coverage attainable with hRNase 4, it is intriguing to speculate that other endoribonucleases that cleave RNA at a similar frequency (on average every eighth nucleotide) may be equivalently useful for RNA LC–MS/MS analysis. A relevant recent study (37), reported the specificity of endoribonuclease toxins from *E. coli*, several of which appear to exhibit di- and mononucleotide specificities. In addition, other endoribonucleases of the RNase A family that exhibit structural and/or sequence homology to hRNase 4 may also display similar specificities. Moreover, thermophilic endoribonucleases that may mimic the cleavage frequency of hRNase 4 would potentially enable cleavage of RNA under high temperature conditions facilitating denaturing of RNA structure. Finally, screening for enzymes that cleave at different combinations of dinucleotide sites is an important area of future development and may yield novel endoribonucleases with useful specificities and further expand the enzymatic toolkit for RNA analysis by LC–MS/MS.

In addition to validating the sequence and modifications present within IVT mRNAs, hRNase 4 will likely be useful for RNA modification mapping in cellular RNAs (e.g. ribosomal RNAs) and in other RNA-based biotechnological platforms, such as RNA-guided CRISPR-Cas genome editing tools. Currently, multiple endoribonucleases have been necessary to achieve robust sequence coverage for modification mapping of native RNA sequences (17) and for sequencing of CRISPR guide RNAs (38). Hence, the use of hRNase 4 may help simplify and improve the specificity of these approaches.

The purified recombinant hRNase 4 enzyme that we have described here may serve applications beyond the analysis of RNA by LC–MS/MS. Given its robust endoribonuclease activity, hRNase 4 may be deployed for the digestion of RNA during DNA and protein purification and in certain enzymatic workflows (for example, reverse transcription), in which efficient RNA digestion may be desirable. Furthermore, in absence of chemical denaturants hRNase 4 may be useful for mapping RNA structural elements. Lastly, due to the close relationship between hRNase 4 and angiogenin (19,21), the RNAs targeted for digestion by hRNase 4 are intriguing yet, altogether undefined. To this end, purified recombinant hRNase 4 may be a valuable tool to help dissect its RNA targets and the molecular underpinnings of its biological activity.

## DATA AVAILABILITY

Relevant code has been made available at https://github.com/ewolf-42/mRNA-Analysis-with-hRNase4. RNA LC–MS/MS data have been deposited to the ProteomeXchange Consortium via the PRIDE partner repository with the dataset identifier PXD033334.

## Supplementary Material

gkac632_Supplemental_File

## References

[1] Damase T.R. , Sukhovershin R., Boada C., Taraballi F., Pettigrew R.I., Cooke J.P. The limitless future of RNA therapeutics. Front. Bioeng. Biotechnol. 2021; 9:628137. 33816449 10.3389/fbioe.2021.628137 PMC8012680

[2] Anderson B.R. , Muramatsu H., Nallagatla S.R., Bevilacqua P.C., Sansing L.H., Weissman D., Karikó K. Incorporation of pseudouridine into mRNA enhances translation by diminishing PKR activation. Nucleic Acids Res. 2010; 38:5884–5892. 20457754 10.1093/nar/gkq347 PMC2943593

[3] Andries O. , Mc Cafferty S., de Smedt S.C., Weiss R., Sanders N.N., Kitada T. N1-methylpseudouridine-incorporated mRNA outperforms pseudouridine-incorporated mRNA by providing enhanced protein expression and reduced immunogenicity in mammalian cell lines and mice. J. Controlled Release. 2015; 217:337–344. 10.1016/j.jconrel.2015.08.051 26342664

[4] Karikó K. , Muramatsu H., Welsh F.A., Ludwig J., Kato H., Akira S., Weissman D Incorporation of pseudouridine into mRNA yields superior nonimmunogenic vector with increased translational capacity and biological stability. Mol. Ther. 2008; 16:1833–1840. 18797453 10.1038/mt.2008.200 PMC2775451

[5] Li B. , Luo X., Dong Y. Effects of chemically modified messenger RNA on protein expression. Bioconjugate Chem. 2016; 27:849–853. 10.1021/acs.bioconjchem.6b00090 26906521

[6] Parr C.J.C. , Wada S., Kotake K., Kameda S., Matsuura S., Sakashita S., Park S., Sugiyama H., Kuang Y., Saito H. N 1-Methylpseudouridine substitution enhances the performance of synthetic mRNA switches in cells. Nucleic Acids Res. 2020; 48:e35. 32090264 10.1093/nar/gkaa070 PMC7102939

[7] Svitkin Y.V. , Cheng Y.M., Chakraborty T., Presnyak V., John M., Sonenberg N. N1-methyl-pseudouridine in mRNA enhances translation through eIF2α-dependent and independent mechanisms by increasing ribosome density. Nucleic Acids Res. 2017; 45:6023–6036. 28334758 10.1093/nar/gkx135 PMC5449617

[8] Muttach F. , Muthmann N., Rentmeister A. Synthetic mRNA capping. Beilstein J. Org. Chem. 2017; 13:2819–2832. 30018667 10.3762/bjoc.13.274 PMC5753152

[9] Nance K.D. , Meier J.L. Modifications in an emergency: the role of N1-Methylpseudouridine in COVID-19 vaccines. ACS Cent Sci. 2021; 7:748–756. 34075344 10.1021/acscentsci.1c00197 PMC8043204

[10] Sutton J.M. , Guimaraes G.J., Annavarapu V., van Dongen W.D., Bartlett M.G. Current state of oligonucleotide characterization using liquid chromatography–mass spectrometry: insight into critical issues. J. Am. Soc. Mass. Spectrom. 2020; 31:1775–1782. 32812756 10.1021/jasms.0c00179

[11] Beverly M. , Dell A., Parmar P., Houghton L. Label-free analysis of mRNA capping efficiency using RNase h probes and LC–MS. Anal. Bioanal.Chem. 2016; 408:5021–5030. 27193635 10.1007/s00216-016-9605-x

[12] Beverly M. , Hagen C., Slack O. Poly a tail length analysis of in vitro transcribed mRNA by LC–MS. Anal. Bioanal. Chem. 2018; 410:1667–1677. 29313076 10.1007/s00216-017-0840-6

[13] Jiang T. , Yu N., Kim J., Murgo J.-R., Kissai M., Ravichandran K., Miracco E.J., Presnyak V., Hua S. Oligonucleotide sequence mapping of large therapeutic mRNAs via parallel ribonuclease digestions and LC–MS/MS. Anal. Chem. 2019; 91:8500–8506. 31129964 10.1021/acs.analchem.9b01664

[14] Addepalli B. , Lesner N.P., Limbach P.A. Detection of RNA nucleoside modifications with the uridine-specific ribonuclease MC1 from momordica charantia. RNA. 2015; 21:1746–1756. 26221047 10.1261/rna.052472.115 PMC4574751

[15] Addepalli B. , Venus S., Thakur P., Limbach P.A. Novel ribonuclease activity of cusativin from cucumis sativus for mapping nucleoside modifications in RNA. Anal. Bioanal. Chem. 2017; 409:5645–5654. 28730304 10.1007/s00216-017-0500-x PMC5709141

[16] Grünberg S. , Wolf E.J., Jin J., Ganatra M.B., Becker K., Ruse C., Taron C.H., Corrêa I.R., Yigit E. Enhanced expression and purification of nucleotide-specific ribonucleases MC1 and cusativin. Protein Expression Purif. 2022; 190:105987. 10.1016/j.pep.2021.105987 34637916

[17] Thakur P. , Estevez M., Lobue P.A., Limbach P.A., Addepalli B. Improved RNA modification mapping of cellular non-coding RNAs using C- and U-specific RNases. Analyst. 2020; 145:816–827. 31825413 10.1039/c9an02111f PMC7002195

[18] Lu L. , Li J., Moussaoui M., Boix E. Immune modulation by human secreted RNases at the extracellular space. Front. Immunol. 2018; 9:1012. 29867984 10.3389/fimmu.2018.01012 PMC5964141

[19] Sheng J. , Luo C., Jiang Y., Hinds P.W., Xu Z., Hu G. Transcription of angiogenin and ribonuclease 4 is regulated by RNA polymerase III elements and a CCCTC binding factor (CTCF)-dependent intragenic chromatin loop. J. Biol. Chem. 2014; 289:12520–12534. 24659782 10.1074/jbc.M114.551762 PMC4007445

[20] Sheng J. , Xu Z. Three decades of research on angiogenin: a review and perspective. Acta Biochim. Biophys. Sin. (Shanghai). 2016; 48:399–410. 26705141 10.1093/abbs/gmv131 PMC4888354

[21] Li S. , Sheng J., Hu J.K., Yu W., Kishikawa H., Hu M.G., Shima K., Wu D., Xu Z., Xin W. et al. Ribonuclease 4 protects neuron degeneration by promoting angiogenesis, neurogenesis, and neuronal survival under stress. Angiogenesis. 2013; 16:387–404. 23143660 10.1007/s10456-012-9322-9 PMC3582744

[22] Cocchi F. , DeVico A.L., Lu W., Popovic M., Latinovic O., Sajadi M.M., Redfield R.R., Lafferty M.K., Galli M., Garzino-Demo A. et al. Soluble factors from t cells inhibiting X4 strains of HIV are a mixture of β chemokines and RNases. Proc. Natl. Acad. Sci. U.S.A. 2012; 109:5411–5416. 22431590 10.1073/pnas.1202240109 PMC3325691

[23] Shapiro R. , Fett J.W., Strydom D.J., Vallee B.L. Isolation and characterization of a human colon carcinoma-secreted enzyme with pancreatic ribonuclease-like activity. Biochemistry. 1986; 25:7255–7264. 3467790 10.1021/bi00371a002

[24] Terzyan S.S. , Peracaula R., de Llorens R., Tsushima Y., Yamada H., Seno M., Gomis-Rüth F.X., Coll M. The three-dimensional structure of human RNase 4, unliganded and complexed with d(up), reveals the basis for its uridine selectivity11Edited by r. Huber. J. Mol. Biol. 1999; 285:205–214. 9878400 10.1006/jmbi.1998.2288

[25] O’Leary N.A. , Wright M.W., Brister J.R., Ciufo S., Haddad D., McVeigh R., Rajput B., Robbertse B., Smith-White B., Ako-Adjei D. et al. Reference sequence (RefSeq) database at NCBI: current status, taxonomic expansion, and functional annotation. Nucleic Acids Res. 2016; 44:D733–D745. 26553804 10.1093/nar/gkv1189 PMC4702849

[26] Henderson J.M. , Ujita A., Hill E., Yousif-Rosales S., Smith C., Ko N., McReynolds T., Cabral C.R., Escamilla-Powers J.R., Houston M.E. Cap 1 messenger RNA synthesis with Co-transcriptional cleancap® analog by in vitro transcription. Curr. Protoc. 2021; 1:e39. 33524237 10.1002/cpz1.39

[27] Zhang Z. , Marshall A.G. A universal algorithm for fast and automated charge state deconvolution of electrospray mass-to-charge ratio spectra. J. Am. Soc. Mass. Spectrom. 1998; 9:225–233. 9879360 10.1016/S1044-0305(97)00284-5

[28] Wein S. , Andrews B., Sachsenberg T., Santos-Rosa H., Kohlbacher O., Kouzarides T., Garcia B.A., Weisser H. A computational platform for high-throughput analysis of RNA sequences and modifications by mass spectrometry. Nat. Commun. 2020; 11:926. 32066737 10.1038/s41467-020-14665-7 PMC7026122

[29] Röst H.L. , Sachsenberg T., Aiche S., Bielow C., Weisser H., Aicheler F., Andreotti S., Ehrlich H.-C., Gutenbrunner P., Kenar E. et al. OpenMS: a flexible open-source software platform for mass spectrometry data analysis. Nat. Methods. 2016; 13:741–748. 27575624 10.1038/nmeth.3959

[30] Mcluckey S.A. , van Berkel G.J., Glish G.L. Tandem mass spectrometry of small, multiply charged oligonucleotides. J. Am. Soc. Mass. Spectrom. 1992; 3:60–70. 24242838 10.1016/1044-0305(92)85019-G

[31] Sato K. , Egami F. The specificity of T1 ribonuclease. C. R. Seances Soc. Biol. Fil. 1957; 151:1792–1796. 13547620

[32] Das U. , Shuman S. Mechanism of RNA 2’,3’-cyclic phosphate end healing by T4 polynucleotide kinase-phosphatase. Nucleic Acids Res. 2013; 41:355–365. 23118482 10.1093/nar/gks977 PMC3592404

[33] Karikó K. , Muramatsu H., Keller J.M., Weissman D Increased erythropoiesis in mice injected with submicrogram quantities of pseudouridine-containing mRNA encoding erythropoietin. Mol. Ther. 2012; 20:948–953. 22334017 10.1038/mt.2012.7 PMC3345990

[34] Thess A. , Grund S., Mui B.L., Hope M.J., Baumhof P., Fotin-Mleczek M., Schlake T. Sequence-engineered mRNA without chemical nucleoside modifications enables an effective protein therapy in large animals. Mol. Ther. 2015; 23:1456–1464. 26050989 10.1038/mt.2015.103 PMC4817881

[35] Mauger D.M. , Cabral B.J., Presnyak V., Su S., Reid D.W., Goodman B., Link K., Khatwani N., Reynders J., Moore M.J. et al. mRNA structure regulates protein expression through changes in functional half-life. Proc. Natl. Acad. Sci. U.S.A. 2019; 116:24075–24083. 31712433 10.1073/pnas.1908052116 PMC6883848

[36] Vaidyanathan S. , Azizian K.T., Haque A.K.M.A., Henderson J.M., Hendel A., Shore S., Antony J.S., Hogrefe R.I., Kormann M.S.D., Porteus M.H. et al. Uridine depletion and chemical modification increase cas9 mRNA activity and reduce immunogenicity without HPLC purification. Mol. Ther. Nucleic Acids. 2018; 12:530–542. 30195789 10.1016/j.omtn.2018.06.010 PMC6076213

[37] Culviner P.H. , Nocedal I., Fortune S.M., Laub M.T. Global analysis of the specificities and targets of endoribonucleases from escherichia coli toxin-antitoxin systems. Mbio. 2021; 12:e0201221. 34544284 10.1128/mBio.02012-21 PMC8546651

[38] Goyon A. , Scott B., Kurita K., Crittenden C.M., Shaw D., Lin A., Yehl P., Zhang K. Full sequencing of CRISPR/Cas9 single guide RNA (sgRNA) via parallel ribonuclease digestions and hydrophilic interaction liquid chromatography–high-resolution mass spectrometry analysis. Anal. Chem. 2021; 93:14792–14801. 34699173 10.1021/acs.analchem.1c03533

