# Supplementary material for: Human RNase 4 improves mRNA sequence characterization by LC–MS/MS
Source: Nucleic Acids Res. 2022 Jul 25;50(18):e106. doi: 10.1093/nar/gkac632 (PMC9561288; doi:10.1093/nar/gkac632)
Supplement: gkac632_Supplemental_File [file gkac632_supplemental_file.pdf]

Human RNase 4 Improves mRNA Sequence Characterization by LC-MS/MS.

Eric J. Wolf, Sebastian Grünberg, Nan Dai, Tien-Hao Chen, Bijoyita Roy, Erbay Yigit, Ivan R. Corrêa Jr.*

New England Biolabs, Inc, 240 County Rd, Ipswich, MA, 01938, USA.

*To whom correspondence should be addressed. Tel: +1 978 380 7504; Email: correa@neb.com

SUPPLEMENTARY DATA

Supplemental Figure S1 related to Figure 1. hRNase 4 cleaves RNA primarily downstream of U and upstream of purines.

Supplemental Figure S2 related to Figure 2. hRNase 4 improves mRNA sequence validation by LC-MS/MS.

Supplemental Figure S3 related to Figure 3. hRNase 4 discriminates between mRNAs modified with $m^1\Psi$ and mo^5U.

Supplementary Figure S4 related to Figure 4. hRNase 4 discriminates between uridine-depleted mRNAs.

Supplementary Table S1. Oligonucleotide templates, reverse transcription primers, and TSO sequences.

Supplementary Table 1. RNA oligonucleotides used in multiplexed pool comprising all possible dinucleotide combinations at least once.

Supplementary Table 2. Endoribonuclease cleavage specificities used in this study.

Supplementary Table 3. RNA oligonucleotides used in multiplexed pool comprising uridine modifications.

Supplementary Table 4. Sequences of IVT mRNAs used in this study.

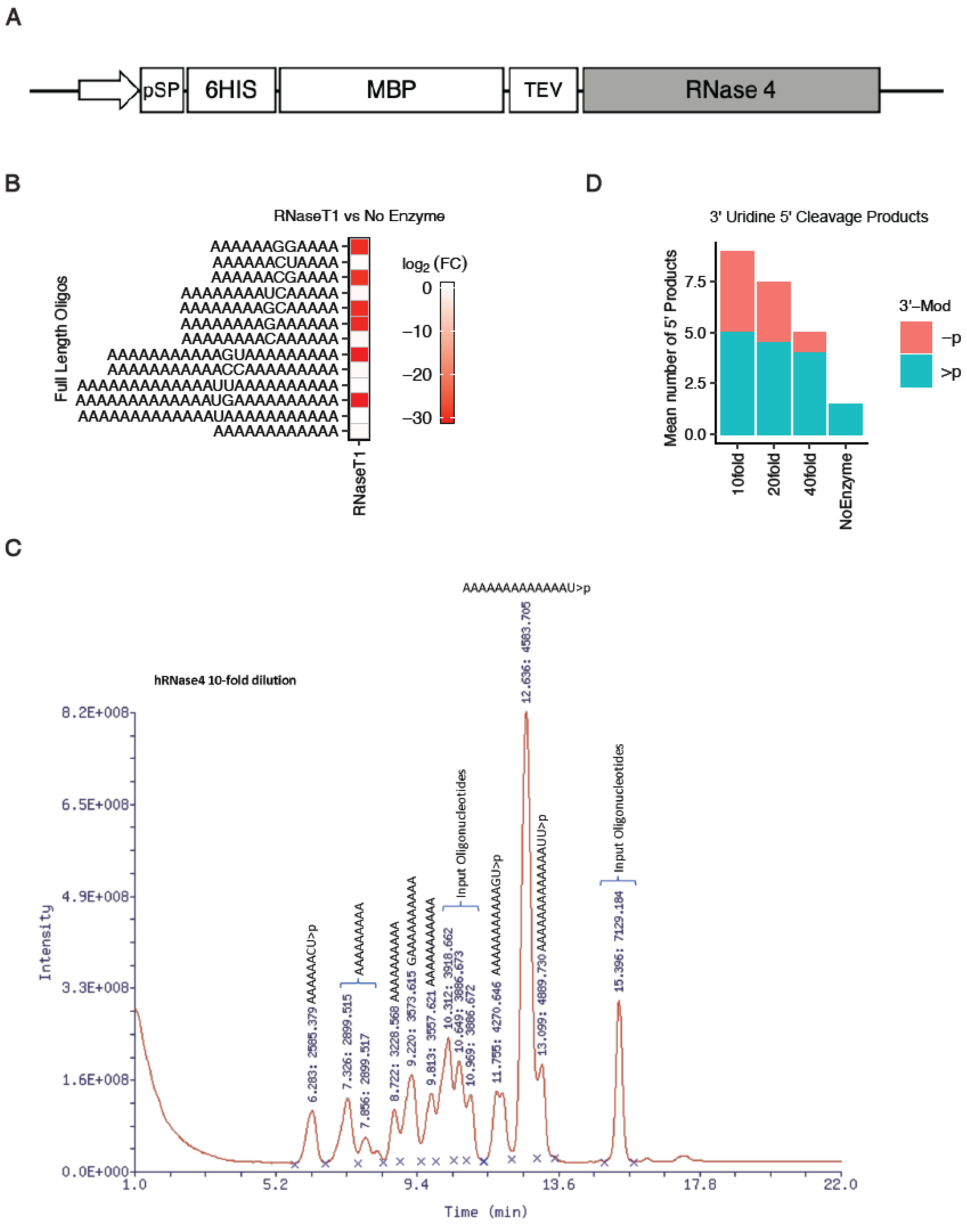

Supplemental Figure S1 related to Figure 1. hRNase 4 cleaves RNA primarily downstream of U and upstream of purines. **(A)** Schematic of the construct utilized for periplasmic expression of hRNase 4 in *E. coli*. **(B)** The mean $\log_2$ fold intensity change for each input oligonucleotide in a multiplexed oligonucleotide pool **(Supplementary Table 1)** after incubation with RNaseT1 relative to experiments performed in the absence of the enzyme. Results are from three independent experiments. **(C)** The mean number of individual 5′ cleavage products with a 3′ terminal uridine comprising either a 2′,3′-cyclic phosphate or 3′-phosphate formed upon incubation of the multiplexed oligonucleotide pool with a dilution series of hRNase 4. Results are from two independent experiments. **(D)** Representative total ion chromatogram from the incubation of the multiplexed oligonucleotide pool with a 1:10 dilution of hRNase 4. The primary mass detected in each chromatographic peak is annotated.

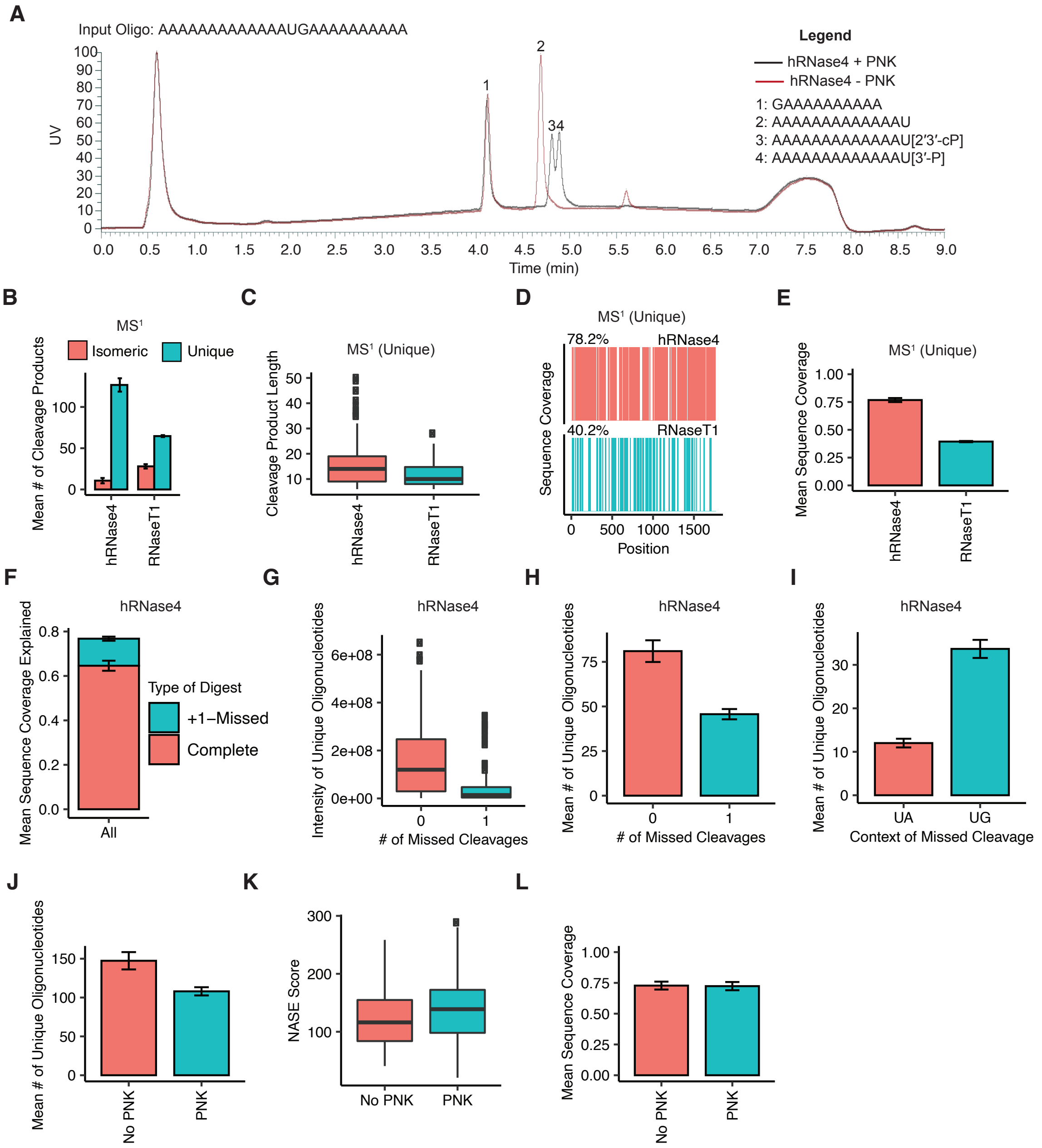

Supplemental Figure S2 related to Figure 2. hRNase 4 improves mRNA sequence validation by LC-MS/MS. **(A)** Overlaid representative UV chromatograms of a single oligonucleotide incubated with hRNase 4 in the presence (*red*) or absence (*black*) of T4 PNK. The primary species present in each chromatographic peak are annotated. **(B)** The mean total number of cleavage products detected by oligonucleotide MS^1 mass analysis (with one missed cleavage) from digestion of fLuc mRNA with either hRNase 4 or RNaseT1. Unique (*green*) and isomeric (*red*) cleavage products are grouped. Error bars represent standard deviation from three independent experiments. **(C)** The length distribution of uniquely mappable cleavage products detected by intact mass analysis from digestion of fLuc mRNA with either hRNase 4 or RNaseT1. **(D)** A sequence coverage map of positions in the fLuc mRNA covered by uniquely mappable cleavage products detected by oligonucleotide MS^1 mass analysis from at least two independent digestions with either hRNase 4 or RNaseT1. The percent sequence

coverage is reported above each map. **(E)** The mean sequence coverage of uniquely mappable cleavage products detected by intact mass analysis from digestion of fLuc mRNA with either hRNase 4 or RNaseT1. Error bars represent standard deviation from three independent experiments. **(F)** The mean sequence coverage explained by uniquely mappable cleavage products with 0 (“Complete”) or a maximum of one missed cleavage (“+1-Missed”) detected by oligonucleotide MS^1 analysis from digestion of fLuc mRNA with hRNase 4. Error bars represent standard deviation from three independent experiments. **(G)** The distribution of intensity of uniquely mappable cleavage products with 0 or 1 missed cleavage events. **(H)** The number of uniquely mappable cleavage products with 0 or 1 missed cleavage events. Error bars represent standard deviation from three independent experiments. **(I)** The number of uniquely mappable cleavage products with one missed cleavage event at either a “UA” or “UG site detected by oligonucleotide MS^1 mass analysis from digestion of fLuc mRNA with hRNase 4. Error bars represent standard deviation from three independent experiments. **(J)** The mean number of distinct oligonucleotides detected by MS/MS-based sequencing (5% FDR) from digestion of fLuc mRNA with hRNase 4 in the presence or absence of T4 PNK. Error bars represent standard deviation from three independent experiments. **(K)** The distribution MS/MS spectral scores as determined by NASE (5% FDR) from digestion of fLuc mRNA with hRNase 4 in the presence or absence of T4 PNK. Replicate digestions were aggregated for this analysis. **(L)** The mean sequence coverage detected by MS/MS-based oligonucleotide sequencing in digestions of fLuc mRNA with hRNase 4 in the presence or absence of T4 PNK. Error bars represent standard deviation from three independent experiments.

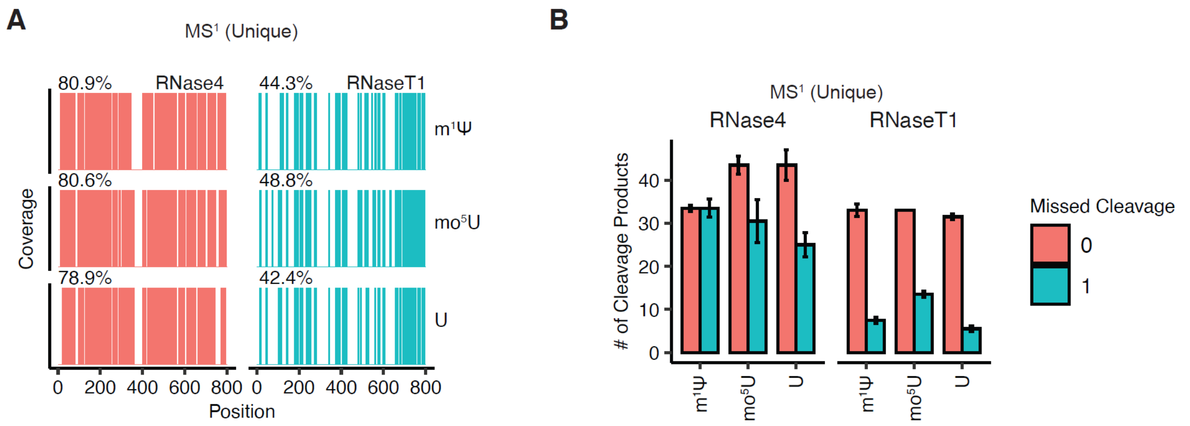

Supplemental Figure S3 related to Figure 3. hRNase 4 discriminates between mRNAs modified with $m^1\Psi$ and mo^5U. (A) A sequence coverage map of positions in the U-, $m^1\Psi$-, or mo^5U-modified EPO mRNA covered by uniquely mappable cleavage products detected by oligonucleotide MS^1 mass analysis from at least two independent digestions with either hRNase 4 or RNaseT1. The percent sequence coverage is reported above each map. (B)The mean total number of cleavage products detected by oligonucleotide MS^1 mass analysis (with one missed cleavage) from digestion of U-, $m^1\Psi$-, or mo^5U-modified EPO mRNA with either hRNase 4 or RNaseT1. Cleavage products with no missed cleavages (g*reen*) or one missed cleavage (*red*) are grouped. Error bars represent standard deviation from two independent experiments.

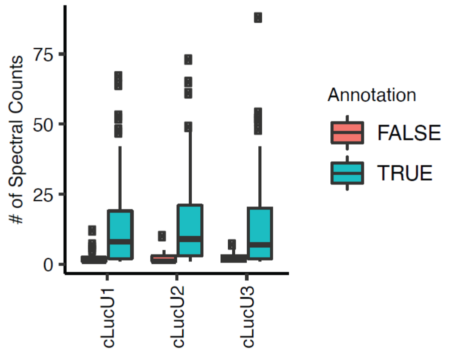

Supplementary Figure S4 related to Figure 4. hRNase 4 discriminates between uridine-depleted mRNAs. The distribution of spectral counts of true (green) and false (red) assigned cleavage products of each uridine-depleted cLuc mRNA from two independent digestions with hRNase 4.

Supplementary Table 1. RNA oligonucleotides used in multiplexed pool comprising all possible dinucleotide combinations at least once.

Dinucleotide(s)	Oligonucleotide Sequence	Source
AA	AAAAAAAAAAAA	IDT
AC/CA	AAAAAAAACAAAAAA	IDT
AG/GA	AAAAAAAAGAAAAAA	IDT
AU/UA	AAAAAAAAAAAAAUAAAAAAAAAAA	IDT
AC/CC/CA	AAAAAAAAAAACCAAAAAAAAA	IDT
AC/CG/GA	AAAAAACGAAAA	IDT
AC/CU/UA	AAAAAACUAAAA	IDT
AG/GC/CA	AAAAAAAAGCAAAAA	IDT
AG/GG/GA	AAAAAAGGAAAA	IDT
AG/GU/UA	AAAAAAAAAAAGUAAAAAAAAA	IDT
AU/UC/CA	AAAAAAAAUCAAAAA	IDT
AU/UG/GA	AAAAAAAAAAAAAUGAAAAAAAAAA	IDT
AU/UU/UA	AAAAAAAAAAAAAUUAAAAAAAAAA	IDT

Supplementary Table 2. Endoribonuclease cleavage specificities used in this study.

Endoribonuclease	Cleaves Between	Reference(s)
Colicin E5	GU	Ogawa et al., 2006
Cusativin-2017	CA, CG, and CU	Addepalli et al., 2017
Cusativin-2021	CG, CU, AU, and UU	Grünberg et al., 2021
MC1-2015	NU	Addepalli et al., 2015
MC1-2021	AU, CU, and UU	Grünberg et al., 2021
RNase A	CN and UN	Volkin and Cohn, 1953
RNase T1	GN	Sato and Egami, 1957
RNase 4	UG and UA	Shapiro et al., 1986; Present Work

Supplementary Table 3. RNA oligonucleotides used in multiplexed pool comprising uridine modifications.

Modification		Oligonucleotide Sequence	Source
-	-	AGAGAGAGAGAGAG	IDT
U	uridine	AAGAGAGAUAGAGAA	IDT
Um	2'-*O*-methyluridine	GAGAGAGAGAGA[Um]AGAGAGAGA	IDT
Ψ	pseudouridine	AGAGAGA[Ψ]AGAG	BioSyn
m^5U	5-methyluridine	GAGAGAGAGA[m^5U]AGAGAGA	BioSyn
m^5Um	5,2'-*O*-dimethyluridine	AGAGAGAGAGA[m^5Um]AGAGAGAG	BioSyn
s^4U	4-thiouridine	AGAGAGAGAGAGA[s^4U]AGAGAGAGAG	BioSyn
D	dihydrouridine	GAGAGAGA[D]AGAGA	TriLink
$m^1\Psi$	1-methylpseudouridine	AGAGAGAGA[$m^1\Psi$]AGAGAG	TriLink

Supplementary Table 4. Sequences of IVT mRNAs used in this study.

mRNA	Input	Sequence
FLuc	10 µg	GGGUCUAGAAAUAAUUUUGUUUAACUUUAAGAAGGAGAUAUAACCA<br>UGAAAAUCGAAGAAGGUAAAGGUCACCAUCACCAUCACCACGGAUC<br>CAUGGAAGACGCCAAAAACAUAAAGAAAGGCCCGGCGCCAUUCUAU<br>CCUCUAGAGGAUGGAACCGCUGGAGAGCAACUGCAUAAGGCUAUGA

		AGAGAUACGCCCUGGUUCCUGGAACAAUUGCUUUUACAGAUGCACA<br>UAUCGAGGUGAACAUCACGUACGCGGAAUACUUCGAAAUGUCCGUU<br>CGGUUGGCAGAAGCUAUGAAACGAUAUGGGCUGAAUACAAAUCACA<br>GAAUCGUCGUAUGCAGUGAAAACUCUCUUCAAUUCUUUAUGCCGGU<br>GUUGGGCGCGUUAUUUAUCGGAGUUGCAGUUGCGCCCGCGAACGA<br>CAUUUAUAAUGAACGUGAAUUGCUCAACAGUAUGAACAUUUCGCAG<br>CCUACCGUAGUGUUUGUUUCCAAAAAGGGGUUGCAAAAAAUUUUGA<br>ACGUGCAAAAAAAAUUACCAAUAAUCCAGAAAAUUAUUAUCAUGGAU<br>UCUAAAACGGAUUACCAGGGAUUUCAGUCGAUGUACACGUUCGUCA<br>CAUCUCAUCUACCUCCCGGUUUUAAUGAAUACGAUUUUGUACCAGA<br>GUCCUUUGAUCGUGACAAAACAAUUGCACUGAUAAUGAAUUCCUCU<br>GGAUCUACUGGGUUACCUAAGGGUGUGGCCCUUCCGCAUAGAACU<br>GCCUGCGUCAGAUUCUCGCAUGCCAGAGAUCCUAUUUUUGGCAAU<br>CAAAUCAUUCCGGAUACUGCGAUUUUAAGUGUUGUUCCAUUCCAUC<br>ACGGUUUUGGAAUGUUUACUACACUCGGAUAUUUGAUAUGUGGAUU<br>UCGAGUCGUCUUAAUGUAUAGAUUUGAAGAAGAGCUGUUUUUACGA<br>UCCCUUCAGGAUUACAAAAUUCAAAGUGCGUUGCUAGUACCAACCC<br>UAUUUUCAUUCUUCGCCAAAAGCACUCUGAUUGACAAAUACGAUUU<br>AUCUAAUUUACACGAAAUUGCUUCUGGGGGCGCACCUCUUUCGAAA<br>GAAGUCGGGGAAGCGGUUGCAAAACGCUUCCAUCUUCCAGGGAUA<br>CGACAAGGAUAUGGGCUCACUGAGACUACAUCAGCUAUUCUGAUUA<br>CACCCGAGGGGGAUGAUAAACCGGGCGCGGUCGGUAAAGUUGUUC<br>CAUUUUUUGAAGCGAAGGUUGUGGAUCUGGAUACCGGGAAAACGC<br>UGGGCGUUAAUCAGAGAGGCGAAUUAUGUGUCAGAGGACCUAUGA<br>UUAUGUCCGGUUAUGUAAACAAUCCGGAAGCGACCAACGCCUUGAU<br>UGACAAGGAUGGAUGGCUACAUUCUGGAGACAUAGCUUACUGGGA<br>CGAAGACGAACACUUCUUCAUAGUUGACCGCUUGAAGUCUUUAAUU<br>AAAUACAAAGGAUAUCAGGUGGCCCCCGCUGAAUUGGAAUCGAUAU<br>UGUUACAACACCCCAACAUCUUCGACGCGGGCGUGGCAGGUCUUC<br>CCGACGAUGACGCCGGUGAACUUCCCGCCGCCGUUGUUGUUUUGG<br>AGCACGGAAAGACGAUGACGGAAAAAGAGAUCGUGGAUUACGUCGC<br>CAGUCAAGUAACAACCGCGAAAAAGUUGCGCGGAGGAGUUGUGUUU<br>GUGGACGAAGUACCGAAAGGUCUUACCGGAAAACUCGACGCAAGAA<br>AAAUCAGAGAGAUCCUCAUAAAGGCCAAGAAGGGCGGAAAGUCCAA<br>ACUCGAGUAAGGUUAACCUGCAGGAGG
EPO	3 µg	GGGGCUUGCUUGUUCUUUUUGCAGAAGCUCAGAAUAAACGCUCAAC<br>UUUGGCACCAUGGGAGUGCACGAGUGUCCCGCGUGGUUGUGGUUG<br>CUGCUGUCGCUCUUGAGCCUCCCACUGGGACUGCCUGUGCUGGGG<br>GCACCACCCAGAUUGAUCUGCGACUCACGGGUACUUGAGAGGUAC<br>CUUCUUGAAGCCAAAGAAGCCGAAAACAUCACAACCGGAUGCGCCG<br>AGCACUGCUCCCUCAAUGAGAACAUUACUGUACCGGAUACAAAGGU<br>CAAUUUCUAUGCAUGGAAGAGAAUGGAAGUAGGACAGCAGGCCGUC<br>GAAGUGUGGCAGGGGCUCGCGCUUUUGUCGGAGGCGGUGUUGCG<br>GGGUCAGGCCCUCCUCGUCAACUCAUCACAGCCGUGGGAGCCCCU<br>CCAACUUCAUGUCGAUAAAGCGGUGUCGGGGCUCCGCAGCUUGAC

GACGUUGCUUCGGGCUCUGGGCGCACAAAAGGAGGCUAUUUCGCC
GCCUGACGCGGCCUCCGCGGCACCCCUCCGAACGAUCACCGCGGA
CACGUUUAGGAAGCUUUUUAGAGUGUACAGCAAUUUCCUCCGCGGA
AAGCUGAAAUUGUAUACUGGUGAAGCGUGUAGGACAGGGGAUCGC
UAGGACUGACUAGGAUCUGGUUACCACUAAACCAGCCUCAAGAACA
CCCGAAUGGAGUCUCUAAGCUACAUAAUACCAACUUACACUUUACA
AAAUGUUGUCCCCCAAAAUGUAGCCAUUCGUAUCUGCUCCUAAUAA
AAAGAAAGUUUCUUCACAUUCUAGCUAG

cLucU1 5 µg GGGAGACCCAAGCUUGGUACCGAGCUCGGAUCCGCCACCAUGAAG
ACCCUGAUCCUGGCCGUGGCCCUGGUGUACUGCGCCACCGUGCAC
UGCCAGGACUGCCCAUACGAACCAGACCCCCCGAACACCGUGCCAA
CCAGCUGCGAGGCCAAGGAAGGCGAGUGCAUCGACAGCAGCUGCG
GCACCUGCACCAGAGACAUCCUGAGCGACGGCCUGUGCGAGAACAA
GCCGGGAAAGACAUGCUGCCGGAUGUGCCAGUACGUGAUCGAGUG
CAGAGUGGAGGCCGCAGGAUGGUUCCGGACCUUCUACGGCAAGAG
AUUCCAGUUCCAAGAGCCCGGCACAUACGUGCUGGGCCAGGGAAC
CAAGGGCGGCGACUGGAAAGUGAGCAUCACCCUGGAGAACCUCGA
CGGCACCAAAGGCGCCGUGCUGACAAAGACAAGACUGGAAGUCGCC
GGCGACAUCAUCGACAUCGCGCAGGCCACCGAGAACCCCAUCACCG
UGAACGGAGGCGCCGACCCCAUAAUCGCCAACCCCUACACAAUCGG
CGAAGUGACAAUCGCCGUCGUGGAAAUGCCAGGCUUCAACAUCACC
GUCAUUGAGUUCUUCAAACUGAUCGUGAUCGACAUCCUCGGAGGAA
GAUCUGUAAGAAUCGCCCCAGACACAGCAAACAAAGGAAUGAUCUC
UGGCCUCUGUGGAGAUCUUAAAAUGAUGGAAGAUACAGACUUCACU
UCAGAUCCAGAACAACUCGCUAUUCAGCCUAAGAUCAACCAGGAGU
UUGACGGUUGUCCACUCUAUGGAAAUCCUGAUGACGUUGCAUACU
GCAAAGGUCUUCUGGAGCCGUACAAGGACAGCUGCCGCAACCCCAU
CAACUUCUACUACUACACCAUCUCCUGCGCCUUCGCCCGCUGUAUG
GGUGGAGACGAGCGAGCCUCACACGUGCUGCUUGACUACAGGGAG
ACGUGCGCUGCUCCCGAAACUAGAGGAACCUGCGUUUUGUCUGGA
CAUACUUUCUACGAUACAUUUGACAAAGCAAGAUACCAAUUCCAGG
GUCCCUGCAAGGAGAUUCUUAUGGCCGCCGACUGUUUCUGGAACA
CUUGGGAUGUGAAGGUUUCACACAGGAAUGUUGACUCUUACACUGA
AGUAGAGAAAGUACGAAUCAGGAAACAAUCGACUGUAGUAGAACUC
AUUGUUGAUGGAAAACAGAUUCUGGUUGGAGGAGAAGCCGUGUCC
GUCCCGUACAGCUCUCAGAACACUUCCAUCUACUGGCAAGAUGGUG
ACAUACUGACUACAGCCAUCCUACCUGAAGCUCUGGUGGUCAAGUU
CAACUUCAAGCAACUGCUCGUCGUACAUAUUAGAGAUCCAUUCGAU
GGUAAGACUUGCGGUAUUUGCGGUAACUACAACCAGGAUUUCAGU
GAUGAUUCUUUUGAUGCUGAAGGAGCCUGUGAUCUGACCCCCAAC
CCACCGGGAUGCACCGAAGAACAGAAACCUGAAGCUGAACGACUCU
GCAAUAGUCUCUUCGCCGGUCAAAGUGAUCUUGAUCAGAAAUGUAA
CGUGUGCCACAAGCCUGACCGUGUCGAACGAUGCAUGUACGAGUA
UUGCCUGAGGGGACAACAGGGUUUCUGUGACCACGCAUGGGAGUU

CAAGAAAGAAUGCUACAUAAAGCAUGGAGACACCCUAGAAGUACCA
GAUGAAUGCAAAUAGGC

cLucU2 5 µg GGGAGACCCAAGCUUGGUACCGAGCUCGGAUCCGCCACCAUGAAG
ACCUUAAUUCUUGCCGUUGCAUUAGUCUACUGCGCCACUGUUCAUU
GCCAGGACUGUCCUUACGAACCUGAUCCACCAAACACAGUUCCAAC
UUCCUGUGAAGCUAAAGAAGGAGAAUGUAUUGAUAGCAGCUGUGGC
ACCUGCACGAGAGACAUACUAUCAGAUGGACUGUGUGAAAAUAAAC
CAGGAAAAACAUGUUGCCGAAUGUGUCAGUAUGUAAUUGAAUGCAG
AGUAGAGGCCGCAGGAUGGUUUAGAACAUUCUAUGGAAAGAGAUUC
CAGUUCCAGGAACCUGGUACAUACGUGUUGGGUCAAGGAACCAAG
GGCGGCGACUGGAAGGUGUCCAUCACCCUGGAGAACCUGGAUGGA
ACCAAGGGGGCUGUGCUGACCAAGACAAGACUGGAAGUGGCUGGA
GACAUCAUUGACAUCGCUCAAGCUACUGAGAAUCCCAUCACUGUAA
ACGGUGGAGCUGACCCUAUCAUCGCCAACCCGUACACCAUCGGCGA
GGUCACCAUCGCUGUUGUUGAGAUGCCAGGCUUCAACAUCACAGU
GAUCGAAUUCUUCAAGCUGAUCGUGAUCGACAUACUGGGCGGACG
GAGCGUGCGCAUCGCCCCAGACACCGCGAACAAGGGCAUGAUCAG
CGGCCUGUGCGGAGACCUGAAGAUGAUGGAGGACACCGACUUCAC
CAGCGACCCCGAGCAGCUGGCCAUCCAGCCAAAAAUCAACCAGGAA
UUCGACGGCUGCCCCCUGUACGGAAACCCCGACGACGUGGCCUAC
UGCAAAGGCCUGCUCGAGCCGUACAAGGACAGCUGCAGAAACCCCA
UCAACUUCUACUACUACACCAUCAGCUGCGCCUUCGCCAGGUGCAU
GGGCGGCGACGAAAGAGCCAGCCACGUCCUGCUGGACUACAGAGA
AACCUGCGCCGCCCCGGAGACACGGGGCACCUGCGUGCUGAGCGG
CCACACCUUCUACGACACAUUCGACAAGGCACGGUACCAGUUCCAG
GGCCCAUGCAAGGAGAUCCUGAUGGCCGCCGACUGCUUCUGGAAC
ACCUGGGACGUGAAGGUGAGCCACAGAAACGUCGACAGCUACACAG
AGGUGGAGAAGGUGAGAAUCAGAAAACAGAGCACAGUGGUGGAACU
GAUCGUGGACGGCAAGCAAAUUCUGGUUGGAGGAGAAGCCGUGUC
CGUCCCGUACAGCUCUCAGAACACUUCCAUCUACUGGCAAGAUGGU
GACAUACUGACUACAGCCAUCCUACCUGAAGCUCUGGUGGUCAAGU
UCAACUUCAAGCAACUGCUCGUCGUACAUAUUAGAGAUCCAUUCGA
UGGUAAGACUUGCGGUAUUUGCGGUAACUACAACCAGGAUUUCAGU
GAUGAUUCUUUUGAUGCUGAAGGAGCCUGUGAUCUGACCCCCAAC
CCACCGGGAUGCACCGAAGAACAGAAACCUGAAGCUGAACGACUCU
GCAAUAGUCUCUUCGCCGGUCAAAGUGAUCUUGAUCAGAAAUGUAA
CGUGUGCCACAAGCCUGACCGUGUCGAACGAUGCAUGUACGAGUA
UUGCCUGAGGGGACAACAGGGUUUCUGUGACCACGCAUGGGAGUU
CAAGAAAGAAUGCUACAUAAAGCAUGGAGACACCCUAGAAGUACCA
GAUGAAUGCAAAUAGGC

cLucU3 5 µg GGGAGACCCAAGCUUGGUACCGAGCUCGGAUCCGCCACCAUGAAG
ACCUUAAUUCUUGCCGUUGCAUUAGUCUACUGCGCCACUGUUCAUU
GCCAGGACUGUCCUUACGAACCUGAUCCACCAAACACAGUUCCAAC
UUCCUGUGAAGCUAAAGAAGGAGAAUGUAUUGAUAGCAGCUGUGGC

		ACCUGCACGAGAGACAUACUAUCAGAUGGACUGUGUGAAAAUAAAC<br>CAGGAAAAACAUGUUGCCGAAUGUGUCAGUAUGUAAUUGAAUGCAG<br>AGUAGAGGCCGCAGGAUGGUUUAGAACAUUCUAUGGAAAGAGAUUC<br>CAGUUCCAGGAACCUGGUACAUACGUGUUGGGUCAAGGAACCAAG<br>GGCGGCGACUGGAAGGUGUCCAUCACCCUGGAGAACCUGGAUGGA<br>ACCAAGGGGGCUGUGCUGACCAAGACAAGACUGGAAGUGGCUGGA<br>GACAUCAUUGACAUCGCUCAAGCUACUGAGAAUCCCAUCACUGUAA<br>ACGGUGGAGCUGACCCUAUCAUCGCCAACCCGUACACCAUCGGCGA<br>GGUCACCAUCGCUGUUGUUGAGAUGCCAGGCUUCAACAUCACCGU<br>CAUUGAGUUCUUCAAACUGAUCGUGAUCGACAUCCUCGGAGGAAGA<br>UCUGUAAGAAUCGCCCCAGACACAGCAAACAAAGGAAUGAUCUCUG<br>GCCUCUGUGGAGAUCUUAAAAUGAUGGAAGAUACAGACUUCACUUC<br>AGAUCCAGAACAACUCGCUAUUCAGCCUAAGAUCAACCAGGAGUUU<br>GACGGUUGUCCACUCUAUGGAAAUCCUGAUGACGUUGCAUACUGCA<br>AAGGUCUUCUGGAGCCGUACAAGGACAGCUGCCGCAACCCCAUCAA<br>CUUCUACUACUACACCAUCUCCUGCGCCUUCGCCCGCUGUAUGGG<br>UGGAGACGAGCGAGCCUCACACGUGCUGCUUGACUACAGGGAGAC<br>GUGCGCUGCUCCCGAAACUAGAGGAACCUGCGUUUUGUCUGGACA<br>UACUUUCUACGAUACAUUUGACAAAGCAAGAUACCAAUUCCAGGGU<br>CCCUGCAAGGAGAUUCUUAUGGCCGCCGACUGUUUCUGGAACACU<br>UGGGAUGUGAAGGUUUCACACAGGAAUGUUGACUCUUACACUGAAG<br>UAGAGAAAGUACGAAUCAGGAAACAAUCGACUGUAGUAGAACUCAU<br>UGUUGAUGGAAAACAGAUCCUGGUGGGCGGCGAAGCCGUGAGCGU<br>GCCAUACAGCAGCCAAAACACCAGCAUCUACUGGCAGGACGGCGAC<br>AUCCUGACAACCGCCAUCCUGCCCGAGGCACUGGUGGUGAAGUUC<br>AACUUCAAACAGCUGCUGGUGGUCCACAUCAGAGACCCCUUCGACG<br>GCAAGACAUGCGGAAUCUGCGGCAACUACAACCAGGACUUCAGCGA<br>CGACAGCUUCGACGCCGAGGGCGCCUGCGACCUGACCCCCAACCC<br>GCCCGGCUGCACCGAGGAACAGAAGCCAGAGGCCGAAAGACUGUG<br>CAACAGCCUCUUCGCCGGACAGAGCGACCUGGACCAGAAGUGCAAC<br>GUGUGCCACAAACCGGACAGAGUGGAACGGUGCAUGUACGAAUACU<br>GCCUGCGGGGCCAGCAGGGAUUCUGCGACCACGCCUGGGAGUUCA<br>AGAAGGAGUGCUACAUCAAGCACGGCGACACCCUGGAGGUGCCAG<br>ACGAGUGCAAGUAGGC
BNT162<br>b2	10 μg	GGAGAAUAAACUAGUAUUCUUCUGGUCCCCACAGACUCAGAGAGAA<br>CCCGCCACCAUGUUCGUGUUCCUGGUGCUGCUGCCUCUGGUGUCC<br>AGCCAGUGUGUGAACCUGACCACCAGAACACAGCUGCCUCCAGCCU<br>ACACCAACAGCUUUACCAGAGGCGUGUACUACCCCGACAAGGUGUU<br>CAGAUCCAGCGUGCUGCACUCUACCCAGGACCUGUUCCUGCCUUU<br>CUUCAGCAACGUGACCUGGUUCCACGCCAUCCACGUGUCCGGCAC<br>CAAUGGCACCAAGAGAUUCGACAACCCCGUGCUGCCCUUCAACGAC<br>GGGGUGUACUUUGCCAGCACCGAGAAGUCCAACAUCAUCAGAGGC<br>UGGAUCUUCGGCACCACACUGGACAGCAAGACCCAGAGCCUGCUGA<br>UCGUGAACAACGCCACCAACGUGGUCAUCAAAGUGUGCGAGUUCCA<br>GUUCUGCAACGACCCCUUCCUGGGCGUCUACUACCACAAGAACAAC

AAGAGCUGGAUGGAAAGCGAGUUCCGGGUGUACAGCAGCGCCAAC
AACUGCACCUUCGAGUACGUGUCCCAGCCUUUCCUGAUGGACCUG
GAAGGCAAGCAGGGCAACUUCAAGAACCUGCGCGAGUUCGUGUUU
AAGAACAUCGACGGCUACUUCAAGAUCUACAGCAAGCACACCCCUA
UCAACCUCGUGCGGGAUCUGCCUCAGGGCUUCUCUGCUCUGGAAC
CCCUGGUGGAUCUGCCCAUCGGCAUCAACAUCACCCGGUUUCAGA
CACUGCUGGCCCUGCACAGAAGCUACCUGACACCUGGCGAUAGCA
GCAGCGGAUGGACAGCUGGUGCCGCCGCUUACUAUGUGGGCUACC
UGCAGCCUAGAACCUUCCUGCUGAAGUACAACGAGAACGGCACCAU
CACCGACGCCGUGGAUUGUGCUCUGGAUCCUCUGAGCGAGACAAA
GUGCACCCUGAAGUCCUUCACCGUGGAAAAGGGCAUCUACCAGACC
AGCAACUUCCGGGUGCAGCCCACCGAAUCCAUCGUGCGGUUCCCC
AAUAUCACCAAUCUGUGCCCCUUCGGCGAGGUGUUCAAUGCCACCA
GAUUCGCCUCUGUGUACGCCUGGAACCGGAAGCGGAUCAGCAAUU
GCGUGGCCGACUACUCCGUGCUGUACAACUCCGCCAGCUUCAGCA
CCUUCAAGUGCUACGGCGUGUCCCCUACCAAGCUGAACGACCUGU
GCUUCACAAACGUGUACGCCGACAGCUUCGUGAUCCGGGGAGAUG
AAGUGCGGCAGAUUGCCCCUGGACAGACAGGCAAGAUCGCCGACU
ACAACUACAAGCUGCCCGACGACUUCACCGGCUGUGUGAUUGCCU
GGAACAGCAACAACCUGGACUCCAAAGUCGGCGGCAACUACAAUUA
CCUGUACCGGCUGUUCCGGAAGUCCAAUCUGAAGCCCUUCGAGCG
GGACAUCUCCACCGAGAUCUAUCAGGCCGGCAGCACCCCUUGUAAC
GGCGUGGAAGGCUUCAACUGCUACUUCCCACUGCAGUCCUACGGC
UUUCAGCCCACAAAUGGCGUGGGCUAUCAGCCCUACAGAGUGGUG
GUGCUGAGCUUCGAACUGCUGCAUGCCCCUGCCACAGUGUGCGGC
CCUAAGAAAAGCACCAAUCUCGUGAAGAACAAAUGCGUGAACUUCA
ACUUCAACGGCCUGACCGGCACCGGCGUGCUGACAGAGAGCAACAA
GAAGUUCCUGCCAUUCCAGCAGUUUGGCCGGGAUAUCGCCGAUAC
CACAGACGCCGUUAGAGAUCCCCAGACACUGGAAAUCCUGGACAUC
ACCCCUUGCAGCUUCGGCGGAGUGUCUGUGAUCACCCCUGGCACC
AACACCAGCAAUCAGGUGGCAGUGCUGUACCAGGACGUGAACUGUA
CCGAAGUGCCCGUGGCCAUUCACGCCGAUCAGCUGACACCUACAU
GGCGGGUGUACUCCACCGGCAGCAAUGUGUUUCAGACCAGAGCCG
GCUGUCUGAUCGGAGCCGAGCACGUGAACAAUAGCUACGAGUGCG
ACAUCCCCAUCGGCGCUGGAAUCUGCGCCAGCUACCAGACACAGAC
AAACAGCCCUCGGAGAGCCAGAAGCGUGGCCAGCCAGAGCAUCAUU
GCCUACACAAUGUCUCUGGGCGCCGAGAACAGCGUGGCCUACUCC
AACAACUCUAUCGCUAUCCCCACCAACUUCACCAUCAGCGUGACCA
CAGAGAUCCUGCCUGUGUCCAUGACCAAGACCAGCGUGGACUGCA
CCAUGUACAUCUGCGGCGAUUCCACCGAGUGCUCCAACCUGCUGC
UGCAGUACGGCAGCUUCUGCACCCAGCUGAAUAGAGCCCUGACAG
GGAUCGCCGUGGAACAGGACAAGAACACCCAAGAGGUGUUCGCCCA
AGUGAAGCAGAUCUACAAGACCCCUCCUAUCAAGGACUUCGGCGGC
UUCAAUUUCAGCCAGAUUCUGCCCGAUCCUAGCAAGCCCAGCAAGC
GGAGCUUCAUCGAGGACCUGCUGUUCAACAAAGUGACACUGGCCG
ACGCCGGCUUCAUCAAGCAGUAUGGCGAUUGUCUGGGCGACAUUG

CCGCCAGGGAUCUGAUUUGCGCCCAGAAGUUUAACGGACUGACAG
UGCUGCCUCCUCUGCUGACCGAUGAGAUGAUCGCCCAGUACACAU
CUGCCCUGCUGGCCGGCACAAUCACAAGCGGCUGGACAUUUGGAG
CAGGCGCCGCUCUGCAGAUCCCCUUUGCUAUGCAGAUGGCCUACC
GGUUCAACGGCAUCGGAGUGACCCAGAAUGUGCUGUACGAGAACC
AGAAGCUGAUCGCCAACCAGUUCAACAGCGCCAUCGGCAAGAUCCA
GGACAGCCUGAGCAGCACAGCAAGCGCCCUGGGAAAGCUGCAGGA
CGUGGUCAACCAGAAUGCCCAGGCACUGAACACCCUGGUCAAGCAG
CUGUCCUCCAACUUCGGCGCCAUCAGCUCUGUGCUGAACGAUAUC
CUGAGCAGACUGGACCCUCCUGAGGCCGAGGUGCAGAUCGACAGA
CUGAUCACAGGCAGACUGCAGAGCCUCCAGACAUACGUGACCCAGC
AGCUGAUCAGAGCCGCCGAGAUUAGAGCCUCUGCCAAUCUGGCCG
CCACCAAGAUGUCUGAGUGUGUGCUGGGCCAGAGCAAGAGAGUGG
ACUUUUGCGGCAAGGGCUACCACCUGAUGAGCUUCCCUCAGUCUG
CCCCUCACGGCGUGGUGUUUCUGCACGUGACAUAUGUGCCCGCUC
AAGAGAAGAAUUUCACCACCGCUCCAGCCAUCUGCCACGACGGCAA
AGCCCACUUUCCUAGAGAAGGCGUGUUCGUGUCCAACGGCACCCA
UUGGUUCGUGACACAGCGGAACUUCUACGAGCCCCAGAUCAUCACC
ACCGACAACACCUUCGUGUCUGGCAACUGCGACGUCGUGAUCGGC
AUUGUGAACAAUACCGUGUACGACCCUCUGCAGCCCGAGCUGGACA
GCUUCAAAGAGGAACUGGACAAGUACUUUAAGAACCACACAAGCCC
CGACGUGGACCUGGGCGAUAUCAGCGGAAUCAAUGCCAGCGUCGU
GAACAUCCAGAAAGAGAUCGACCGGCUGAACGAGGUGGCCAAGAAU
CUGAACGAGAGCCUGAUCGACCUGCAAGAACUGGGGAAGUACGAG
CAGUACAUCAAGUGGCCCUGGUACAUCUGGCUGGGCUUUAUCGCC
GGACUGAUUGCCAUCGUGAUGGUCACAAUCAUGCUGUGUUGCAUG
ACCAGCUGCUGUAGCUGCCUGAAGGGCUGUUGUAGCUGUGGCAGC
UGCUGCAAGUUCGACGAGGACGAUUCUGAGCCCGUGCUGAAGGGC
GUGAAACUGCACUACACAUGAUGACUCGAGCUGGUACUGCAUGCAC
GCAAUCCUAGCUGCCCCUUUCCCGUCCUGGGUACCCCGAGUCUCC
CCCGACCUCGGGUCCCAGGUAUGCUCCCACCUCCACCUGCCCCAC
UCACCACCUCUGCUAGUUCCAGACACCUCCCAAGCACGCAGCAAUG
CAGCUCAAAACGCUUAGCCUAGCCACACCCCCACGGGAAACAGCAG
UGAUUAACCUUUAGCAAUAAACGAAAGUUUAACUAAGCUAUACUAAC
CCCAGGGUUGGUCAAUUUCGUGCCAGCCACACCCUGGACCUAGCG
CGGCCGGCUAG
